# Anti-Tumorigenic Effects of Sea Buckthorn Root Extracts on Head and Neck Cancer Cells—A Systematic Analysis

**DOI:** 10.3390/ijms26104625

**Published:** 2025-05-12

**Authors:** Alina Gazizova, Manuel Gronbach, Christina Oppermann, Udo Kragl, Nadja Engel

**Affiliations:** 1 Department of Industrial and Analytical Chemistry, Institute of Chemistry, University of Rostock, Albert-Einstein-Straße 3a, 18059 Rostock, Germany; alina.gazizova@uni-rostock.de (A.G.); manuelgronbach@web.de (M.G.); christina.oppermann@uni-rostock.de (C.O.); udo.kragl@uni-rostock.de (U.K.); 2 Department of Life, Light and Matter, University of Rostock, Albert-Einstein-Straße 25, 18059 Rostock, Germany; 3 Department of Oral, Maxillofacial and Plastic Surgery, University Medicine Rostock, Schillingallee 35, 18057 Rostock, Germany

**Keywords:** anticancer drug, screening, head and neck cancer, LC-MS, NMR

## Abstract

Chemotherapy is a common treatment method for cancer that is often associated with strong side effects. To reduce these, research on extracts from medicinal plants and their active ingredients has been conducted. Although sea buckthorn (*Hippophae rhamnoides*) is a well-established medicinal plant, little is known about the chemical components responsible for its putative anticancer activity. This study focuses on both chemical and medical analyses of methanolic sea buckthorn root extracts. Cell viability measurements were performed on head and neck cancer cell lines, as well as non-tumorigenic control cells. Microwave and classical extractions under reflux were used to prepare the methanolic extracts. LC/MS and NMR were used to determine the structures of the molecules contained within these extracts. The aqueous phase of one sea buckthorn root extract reduced the viability of cancer cells, whereas the viability of non-tumorigenic control cells remained unaltered. The cell cycle phases of cancer cells treated with the extract shifted in comparison to control treatment. After 24 h, the number of cells in proliferative phases had increased. Two fractions of the extract that evoked alterations were identified. After a 48 h treatment, one of the fractions showed a higher number of apoptotic cells than the control. LC/MS and NMR analyses were conducted to attempt to identify the active compounds. We propose that the bioactivity of this extract is caused by a mixture of 2′-hydroxyflavone isomers.

## 1. Introduction

Cancer is one of the leading causes of death worldwide, causing nearly 10,000,000 deaths in 2020. Nearly 200,000 of those were lip cancers and 50,000 were oral cavity cancers, with almost 400,000 and 100,000 new cases identified, respectively [1]. The treatment for these cancers usually involves radiotherapy or chemotherapy. Both have side effects, some of which might be severe, long-term, or even permanent. For example, radiotherapy may permanently alter or damage mucous membranes and salivary glands, causing permanent dry mouth and difficulty in eating, swallowing, and speaking [2]. Common side effects of chemotherapy are hair loss and nausea, as well as mouth and throat sores [3]. Most are caused by the cytotoxic effects of cytostatic drugs, which affect not only tumorigenic but also non-tumorigenic cells [4].

To develop drugs that are less toxic to non-tumorigenic cells, because they either specifically target tumor cells or protect non-tumorigenic cells, investigations into plants and their naturally occurring constituents are very worthwhile. Since plants have been used in medicine for centuries, there is an interest in scientifically analyzing their constituents and identifying their active ingredients. The four types of plant-derived substance classes commonly used in chemotherapy are vinca alkaloids, epipodophyllotoxins, taxanes, and camptothecin [5]. Paclitaxel, which is one of the most frequently used chemotherapeutic drugs, belonging to the taxane class, was first isolated from pacific yew in 1971 [6].

One of the plants that has recently been analyzed is sea buckthorn (Hippophae rhamnoides). It has been described to have anti-inflammatory [7,8,9], antioxidative [10], anti-stress [9], hepatoprotective [11], and cardioprotective [12] properties. Recently, its influence on cancer cells has been investigated. A study showed that isorhamnetin, which was isolated from *Hippophae rhamnoides* L., displayed anticancer activity on the cell line BEL-7402 [13]. Another study described the inhibition of the colon cancer cell line HT-29 and breast cancer cell line MCF-7 by sea buckthorn extracts [14]. Its berry extracts were found to inhibit the proliferation of Caco-2 cells and cause apoptosis in Caco-2 and Hep-G2 cells [15]. It was further shown that treatment with ethanolic berry extracts caused alterations in MCF-7 metabolism; changes in acidification and respiration were observed [16].

Despite investigations into the chemical composition and biological activity of sea buckhorn fruit, seeds, and leaves, little is known about the substances contained within sea buckthorn root (SBR). In a study of Korean sea buckthorn, it was found that the root contained catechin, isorhamnetin, and rutin [17], and, according to another study, compounds found in SBR such as syringetin, isorhamnetin, and pentamethylquercetin are able to inhibit tumor cell proliferation. More importantly, quercetin and myricetin even have an apoptotic effect on cancer cells while not affecting non-tumorigenic cells [18]. However, it is noteworthy that no publication in which the effects of SBR extracts were directly tested on cells could be found.

Therefore, the aim of this study was to further analyze the composition of SBR extracts and conduct in vitro experiments on head and neck cancer cells. To achieve these aims we used column chromatography, liquid chromatography/mass spectrometry (LC/MS), and nuclear magnetic resonance spectrometry (NMR) for separation and chemical analysis, as well as a metabolic activity assay, flow cytometry, and ECIS for in vitro testing.

## 2. Results and Discussion

### 2.1. Metabolic Activity Assay

**Figure 1:**
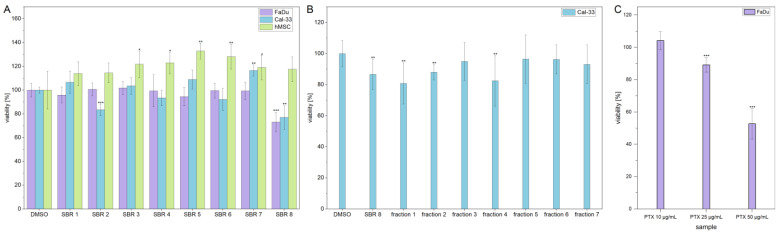
(**A**) Viability of FaDu (violet), Cal-33 (blue), and hMSCs (green) after 24 h treatment with SBR extracts. (**B**) Viability of Cal-33 after treatment with SBR 8 and its fractions, obtained through column chromatography. (**C**) Viability of FaDu after 24 h treatment with different concentrations of Paclitaxel (PTX). Mean ± SD with n = 6, * *p* < 0.05, ** *p* < 0.01, *** *p* < 0.001.

MTS assays were performed with aqueous phases of SBR extracts on two cancer cell lines (FaDu and Cal-33) and hMSCs, which were used as a non-tumorigenic control cell line. The results are displayed in Figure 1A. The viability of FaDu and Cal-33 decreased by approximately 20% after treatment with SBR 8. Interestingly, hMSC control cells were not affected by the treatment. In addition, the viability of Cal-33 treated with SBR 2 also decreased by 20%. However, since this extract did not seem to affect FaDu in the same way, it was not further investigated.

**Figure 2:**
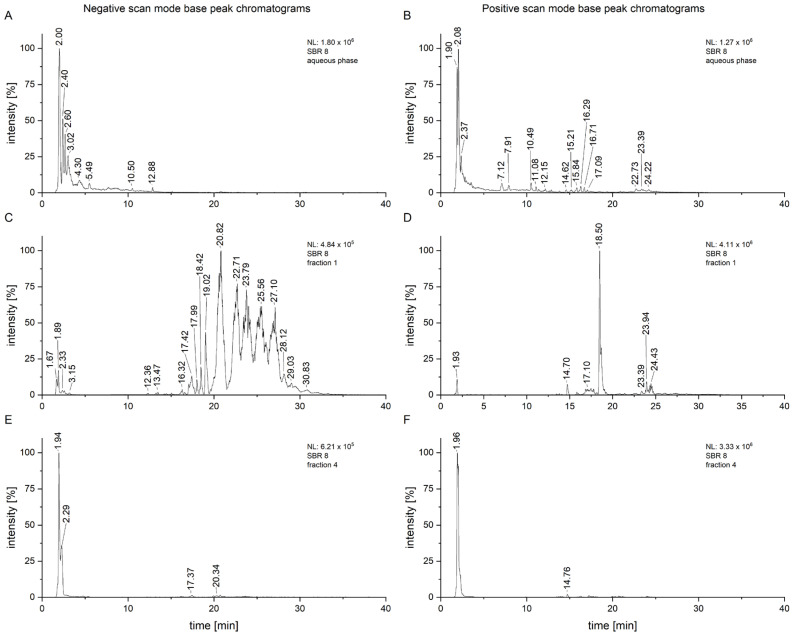
Negative- and positive-scan-mode chromatograms of the aqueous phase of SBR 8 (**A**,**B**) and its fractions 1 (**C**,**D**) and 4 (**E**,**F**).

SBR 8 was separated into seven fractions using column chromatography, which were then tested on Cal-33. A moderate viability reduction was observed with fractions 1 and 4. A comparison of the chromatograms of SBR 8 (in its aqueous phase) and its fractions 1 and 4 is displayed in Figure 2.

**Figure 3:**
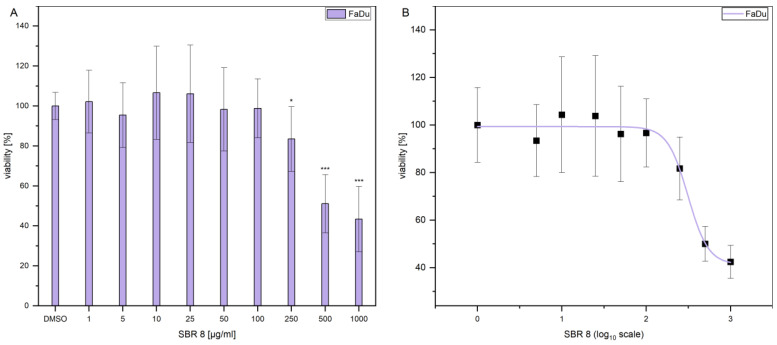
(**A**) MTS assay results after 24 h treatment of FaDu with different concentrations of SBR 8. (**B**) MTS assay results after 24 h treatment of FaDu with different concentrations of SBR 8, plotted on a logarithmic scale for LD50 determination. Mean ± SD with n = 6, * *p* < 0.05, *** *p* < 0.001.

Additionally, the LD_50_ was established by performing dose response experiments on FaDu (Figure 3A). The results indicate that a significant reduction in FaDu viability could only be achieved at higher extract concentrations. Figure 3B displays the logarithmic plot used to determine the LD_50_. The LD_50_ value was calculated to be 299.74 µg/mL. Since the extract reduced the viability only moderately and contained multiple compounds (Figure 2A,B), with not all of them having a reducing effect on cell viability (Figure 1B), it is logical that a higher concentration of the extract is needed to reduce the viability of the cells by 50%. As a comparison, Figure 1C shows that treatment with Paclitaxel, an established chemotherapeutic agent, results in a decrease in the viability of FaDu at significantly lower concentrations. The determined LD50 values for the SBR 8 extract are not physiological and, therefore, lack clinical relevance. This can be attributed to the fact that the extract comprises a mixture of constituents, not all of which exhibit anti-tumorigenic potential. Consequently, the primary objective of fractionation is to identify its active compounds. It is important to note that individual substances may exhibit different activity profiles after purification compared to their activity within the natural composite matrix.

As displayed in Figure 2, some of the peaks are much more intensive in the fractions compared to the chromatogram of the whole aqueous phase, especially in fraction 1 (Figure 2C,D). This fraction had a small yield overall, but the measurements were conducted with a concentration of 1 mg/mL, allowing for a much clearer analysis. It is also clear that the fraction still represents a mixture of compounds which would have to be further separated for proper isolation. This, however, also suggests that low concentrations of an active compound can still have effects on cancer cells. Fraction 4, which appears to consist of only one substance, still shows more peaks upon closer inspection, some of which are consistent with those from fraction 1 (Figure 2E,F).

### 2.2. Cell Cycle Analysis

**Figure 4:**
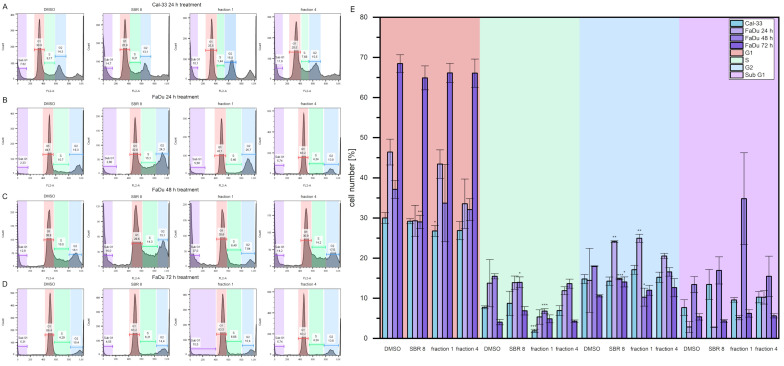
Example histograms of flow cytometry measurements of (**A**) Cal-33 after 24 h treatment and (**B**–**D**) FaDu after 24, 48, and 72 h treatments. (**E**) Statistical analysis of flow cytometry measurement results. Mean ± SD with n = 3, * *p* < 0.05, ** *p* < 0.01, *** *p* < 0.

Cell cycle analyses were performed to validate the MTS assay results. Figure 4A–D show histogram examples of Cal-33 (A) and FaDu (B, C, and D for 24, 48, and 72 h, respectively) treated with DMSO, SBR 8 fraction 1, and SBR and fraction 4. Figure 4E presents the cell cycle distribution. Cells treated with SBR 8 and its fractions were compared to cells treated with DMSO.

Cal-33 cells treated with SBR 8 for 24 h display a slightly higher number of cells in the Sub G1 phase (13.5%) compared to cells treated with DMSO (7.7%). Treatment with fraction 1 revealed a lower number of cells in the G1 and S phases (26.8% and 1.9%, respectively) in comparison to DMSO (30.0% and 7.8%, respectively), whereas the G1 and Sub G1 phases remained comparable to those seen with DMSO. Fraction 4 did not seem to affect Cal-33 after 24 h of treatment.

FaDu treated with SBR 8 for 24 h had a slightly lower number of cells in its G1 phase (29.3%) in comparison to DMSO (46.4%) and a significantly higher number of cells in its G2 phase (24.1%, DMSO: 14.5%). A similar result was also observed after treatment with fraction 1, where the number of cells in the G2 phase was also significantly higher (25.0%). Fraction 1 did not seem to affect the G1 phase; however, it did affect the number of cells in the S phase, which was lowered from 13.8% (DMSO) to 5.3%. A lower number of cells in the G1 phase (33.5%) and higher number of cells in the Sub G1 (10.3%, DMSO: 2.9%) phase was observed after a 24 h treatment with fraction 4.

After the 48 h treatment, the cell cycle changes in FaDu became more evident. The cell numbers of the G1, S, and G2 phases dropped significantly after treatment with SBR 8 (29.0, 14.0, and 14.8%, respectively) in comparison to DMSO (37.1, 15.5, and 18.0%, respectively) and the number of cells in the Sub G1 phase increased significantly, from 13.4% (DMSO) to 16.9%. Treatment with fraction 1 showed the same changes to all cell cycle phases (33.7% G1, 6.8% S, 10.3% G2, 34.8% Sub G1). Fraction 4 also slightly lowered the number of cells in the G1 phase (32.0%). However, the 72 h treatment of FaDu did not reveal any significant changes in the cell cycle.

**Figure 5:**
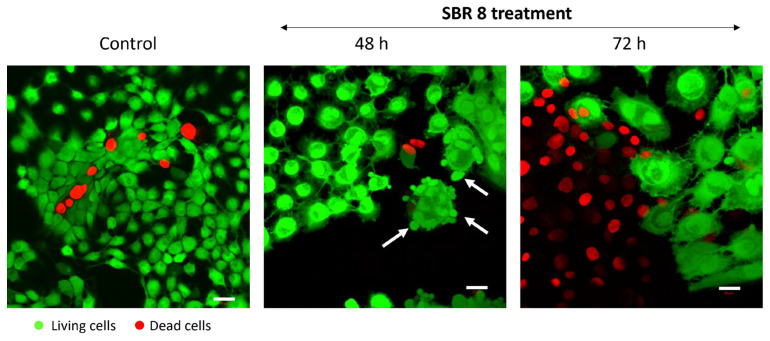
Live/dead cell staining using a green fluorophore to label viable cells and a red probe to indicate non-viable cells. Under control conditions, Cal-33 cells show a minimal presence of dead cells. In contrast, treatment with SBR 8 leads to a significant increase in apoptotic cells after 72 h. Signs of apoptosis, including membrane blebbing (indicated by white arrows) and swollen cell bodies, are already apparent after 48 h of treatment. The white scale bar represents 20 µm.

It is evident that SBR 8 alters the cell cycle of Cal-33 and FaDu. The Sub-G1 phase serves as a strong indicator of apoptosis, while the S and G2 phases are associated with proliferative activity. After 24 h of treatment with SBR 8, the number of cells in the Sub-G1 phase increased in Cal 33, indicating the presence of apoptotic cells. In contrast, the number of FaDu cells in the G2 phase significantly increased in accordance with the lowered number of cells in the G1 phase. The number of cells in the S phase was also higher in FaDu. After the 48 h treatment of FaDu with SBR 8, the numbers of cells in all three proliferative phases were significantly lower than in the DMSO control and the number of cells in the Sub G1 phase was higher. This indicates a cell cycle arrest in the G2 phase (24 h treatment) and subsequent apoptosis (48–72 h), which was further confirmed through fluorescence imaging (Figure 5). Apoptosis induction becomes apparent after just 48 h of treatment, as evidenced by membrane blebbing (white arrows) and swollen cell bodies. By 72 h, the proportion of apoptotic cells increases substantially, particularly in the periphery of the cell clusters most sensitive to the treatment.

Similarly, in a study of *Rheum turkestanicum*, it was found that the viability of HeLa and MCF-7 decreased noticeably after 48 and 72 h treatments with the root extract, as opposed to treatment for only 24 h [19]. During the 72 h treatment, SBR 8 did not appear to alter the cell cycle of FaDu significantly. This could be due to the low extract concentration used, since the applied concentration was lower than the calculated LD50. Cells not impacted by the extract could still proliferate, leading to the seemingly normal cell cycle. Even though no significant changes were observed, the trends observed after the 24 h treatment were also visible after the 72 h treatment (Figure 4D). Fraction 1 seemed to slightly raise the number of cells in the G2 phase, while Fraction 4 seemed to raise the number of cells in the S phase. SBR 8 seemed to slightly raise both phases. It is also noteworthy that Paclitaxel, a drug commonly used in chemotherapeutic cancer treatment, shows similar results, inducing cell cycle arrest in either the G1 or G2/M phase without leading to apoptosis, when applied at low concentrations [20].

### 2.3. Real-Time Electric Cell-Substrate Impedance Sensing

**Figure 6:**
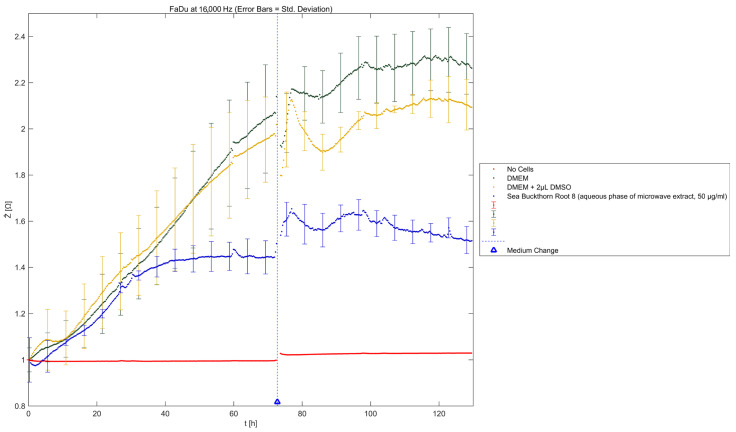
Results of ECIS measurements; normalized impedance (Ẑ) as a function of time (t). Mean ± SD with n = 3.

The effects of SBR 8 on FaDu were additionally observed via ECIS (Figure 6). The normalized impedance represents the coverage of the electrode by adhered cells. It rises with the attached cell number.

As is visible in Figure 6, the impedance signal of cells treated with SBR 8 rose similarly to that of cells treated with DMSO (yellow) for approximately 40 h. Then, while the impedance signal kept rising for DMSO-treated cells, that of the cells treated with SBR 8 started stagnating and remained at the same level. Even after the medium was changed to extract-free medium at 72 h, the signal did not increase but instead remained stagnant.

These observations correspond to the FACS results, showing that significant changes can be seen after over 40 h of treatment. A low impedance signal corresponds to a low electrode coverage. This could indicate apoptosis as cells detach during apoptosis. However, it is also possible that the cells were arrested in their growth without detaching. Impedance not rising after the change to extract-free medium suggests that the cells did not return to their usual proliferation cycle. Therefore, the extract seemed to fundamentally alter the cells. This supports the hypothesis that the extract induces cell cycle arrest.

### 2.4. Chemical Analysis of SBR 8 Extract

**Figure 7:**
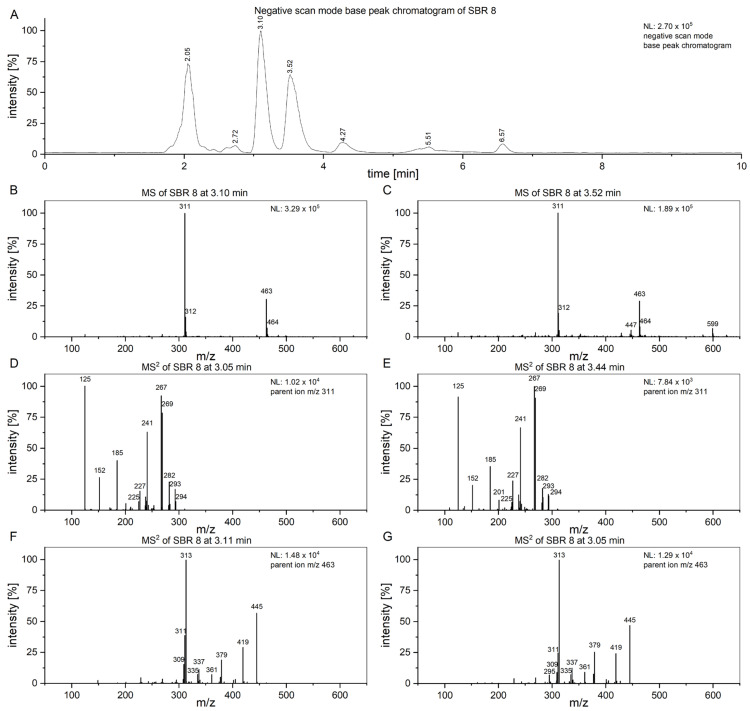
(**A**) Chromatogram of SBR 8’s aqueous phase, measured using Method B. (**B**,**C**) MS of peaks at 3.10 and 3.44 min. (**D**,**E**) MS^2^ spectra of parent ion at *m*/*z* 463. (**F**,**G**) MS^2^ spectra of parent ion at *m*/*z* 463.

To attempt to find compounds possibly responsible for the observed biomedical effect, an LC/MS^2^ analysis of SBR 8 (Figure 7) was performed. In Figure 7A, the negative-scan-mode chromatogram of the aqueous phase of SBR 8 is shown. The peaks at 3.10 and 3.52 min have similar mass spectra (Figure 7B,C) and the peaks with *m*/*z* 311 and 463 are also found in fraction 1 and fraction 4. Figure 7D,E show the MS^2^ of the peaks at 3.10 and 3.52 min with an *m*/*z* 311 parent ion.

The spectra are nearly identical at both retention times, indicating that the substances represented by the peaks are structurally identical. Figure 7F,G show the spectra of the peaks at the same time when using an *m*/*z* 463 parent ion. They are also nearly identical. Therefore, the peaks at 3.10 and 3.52 min must both contain two substances.

**Figure 8:**
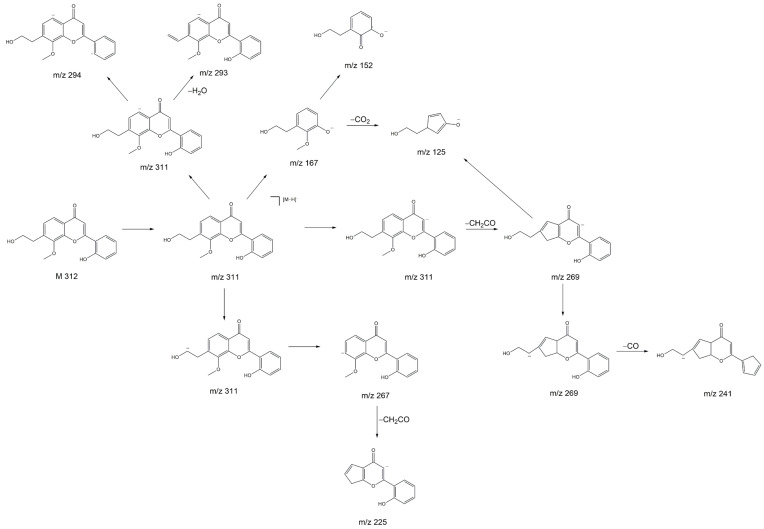
Proposed fragmentations of a 7-(2-hydroxyethyl)-8-methoxy-2′-hydroxyflavone.

The product ion spectrum of the parent ion at *m*/*z* 311 shows particularly intensive fragment ion peaks at *m*/*z* 267 and *m*/*z* 125, which is typical for 2′-hydroxyflavonols [21]. We propose that the substance with the molecular ion peak at *m*/*z* 311 belongs to 7-(2-hydroxyethyl)-8-methoxy-2′-hydroxyflavone. Despite not being a hydroxyflavonol, the substance has a very similar structure to a 2′-hydroxyflavonol. The proposed fragmentation of 7-(2-hydroxyethyl)-8-methoxy-2′-hydroxyflavone is displayed Figure 8 as an example of a substance which could yield the fragments observed in Figure 7D,E. It is noteworthy that we see two peaks with slightly different retention times and nearly identical fragmentation patterns in Figure 7. This indicates the presence of very structurally similar substances that contain only a slight difference, e.g., in their methoxy group placement.

The anticancer and antioxidative properties of several 2′-hydroxyflavone derivatives have previously been established, which explains the results we achieved during our tests on cancer cells. Further, it has been shown that 3,5,7 trimethodyflavone, a structurally similar substance, has a positive effect on normal human dermal fibroblasts, protecting cells against reactive oxygen species damage [22]. This might be correlated with the positive proliferative effects of SBR 8 on hMSCs (Figure 1A).

The substance at *m*/*z* 463 was not identified. Since its retention time is similar to 7-(2-hydroxyethyl)-8-methoxy-2′-hydroxyflavone, it must have similar chemical properties. The compound with an *m*/*z* of 463 does not display a peak with an *m*/*z* of 301 (Figure 7F,G), which would be attributed to the loss of a hexoside, as is typical for quercetin derivatives such as spiraeoside or isoquercetin [23]. The fragment with *m*/*z* 300, which is commonly seen in flavonoids as well, was also not observed here [24]. Further, the fragment ion spectrum of the compound with *m*/*z* 463 does not yield a fragment ion with *m*/*z* 311, indicating that the substance with *m*/*z* 463 is not a 7-(2-hydroxyethyl)-8-methoxy-2′-hydroxyflavone derivative. The fragment ion with *m*/*z* 445 can be attributed to H2O loss and the fragment with *m*/*z* 419 is likely caused by CO2 loss.

In the chromatogram of fraction 4, the most intensive peaks are found at 1.93 and 2.24 min. However, the mass spectrum at 1.93 min shows a peak at *m*/*z* 341. We attributed it to sucrose, because we were able to isolate sucrose from SBR using column chromatography (the relevant NMR and LC/MS spectra can be found in Supplementary Materials). The peak at 2.24 min has an *m*/*z* at 377. This was further analyzed using MS^2^. It yielded a fragment ion at *m*/*z* 341, which was attributed to sucrose. We suggest that the parent ion is an adduct of sucrose and chloride [25]. The rest of fraction 4 is similar to fraction 1. Since sucrose did not cause the MTS assay results, it is most likely that the rest of fraction 4, which is the same as the contents of fraction 1, contains the bioactive substances.

We also analyzed two peaks in the positive-scan-mode chromatograms via MS^2^ (with parent ions at *m*/*z* 365 and 448). The mass spectra can be viewed in the Supplementary Materials.

## 3. Materials and Methods

### 3.1. Chemicals

For extraction and separation via column chromatography, HPLC-grade chemicals (methanol (MeOH), ethyl acetate (EtOAc)), purchased from Fisher Chemicals (Fisher Scientific GmbH, Schwerte, Germany), were used. Solvents for the LC/MS analysis (LC/MS quality) were obtained from Fisher Chemicals (Fisher Scientific GmbH, Schwerte, Germany) and Honeywell Deutschland Holding GmbH, Offenbach, Germany. The silica gel (technical grade, pore size 60 Å, 230–400 mesh particle size, 40–63 pm particle size) used for column chromatography was purchased from Sigma-Aldrich (Sigma-Aldrich Chemie GmbH, Schnelldorf, Germany). DMSO with a purity of ≥99.0%, which was used as a solvent in the cell tests, was purchased from VWR Chemicals (VWR International GmbH, Darmstadt, Germany). The MTS reagent used for assays was acquired from Promega (CellTiter 96^®^ AQueous One Solution Cell Proliferation Assay, Promega GmbH, Walldorf, Germany).

### 3.2. Plant Material

Eight sea buckthorn root samples of the “Habego” variety were obtained from the Research Institute for Agriculture and Fishery of Mecklenburg-Western Pomerania (Germany). There, cuttings from the previous year, obtained from a Baltic Sea tree farm in Kröpelin (Germany), were planted on 4 April 2020 and grown in varying soils and under different conditions. A list of these conditions is provided in the Supporting Information. The cultivation, harvesting, and experimental research into the sea buckthorn variety “Habego” comply with the relevant institutional, national, and international guidelines and legislation. All experiments comply with The International Union for Conservation of Nature (IUCN) Policy Statement and were not indexed to the IUCN red list index of threatened species. Voucher specimens were deposited in a public herbarium of the Department of Biological Sciences and the Department of Botany and Botanical Garden Institute of Biological Sciences, University of Rostock, Rostock, Germany. The plants were identified by Dr. Frank Hippauf and reference specimens were deposited at the State Research Institute for Agriculture and Fishery of Mecklenburg-West Pomerania, Gülzow-Prüzen, Germany.

### 3.3. Plant Material Preparation

Root material was washed thoroughly using tap water and rinsed with ultra-pure water. Frankia actinomycetes were removed from the stem. The roots were cut using gardening shears to reduce their size, dried via lyophilization (VaCo 2, Zirbus technology GmbH, Bad Grund, Germany), and ground using a laboratory mill (IKA^®^ Werke, Staufen im Breisgau, Germany) with a maximum particle size of 1 mm.

### 3.4. Extraction

Plant powders were extracted via microwave extraction: 4.5 g plant powder was separated into 3 PFA vessels. We added 13.5 mL of MeOH to each tube. The powders were extracted 3 times using fresh MeOH. CEM MarsXpress™ (Kamp-Lintfort, Germany) was used, and the conditions were set to 60 °C and 400 MW (5 min warming, 10 min constant heating, 5 min cooling). After each extraction step, the liquid phase was carefully decanted and filtered using a Millipore^®^ system and a Durapore filter (pore size 0.22 µm). A 1 mL sample was taken for LC/MS measurements and MeOH was removed in vacuo. The dried extract was dissolved in 20 mL of water and extracted with 10 mL of EtOAc a total of 3 times. The phases were separated, yielding an EtOAc and an aqueous phase. Water was removed via freeze drying (VaCo 2, Zirbus technology GmbH, Bad Grund, Germany); ethyl acetate was removed via a rotary evaporator.

### 3.5. Column Chromatography

The separation of 0.2 g of the aqueous phase of SBR 8 was performed on silica gel at an air pressure of 4 psi. As a mobile phase, the following gradients of ethyl acetate and MeOH (respectively) were used: 100:50 and 100:100. Acetic acid (1%) was added to each eluent.

Fractions were collected in test tubes and TLC was used to determine the nature of the fractions. A solution of anis aldehyde in ethanol (solution recipe in Supplementary Materials) was used to stain the spots. Individual fractions were combined in separate flasks and the solvent was removed in vacuo, yielding 7 fractions. The fractions were dissolved in DMSO and used for biomedical testing after a sample was taken for LC/MS analysis.

### 3.6. LC/MS and LC/MS^2^ Analysis

Extract analysis was performed using LC/MS. The system consisted of an UltiMate™ 3000 liquid chromatograph coupled with an LTQ XL™ mass spectrometer (both obtained from Thermo Fisher Scientific, Schwerte, Germany). The LC was equipped with a Kinetex^®^ phenyl-hexyl (150 × 2.1 mm × 2.6 µm) column (Phenomenex^®^, Aschaffenburg, Germany) and the eluent consisted of MeOH and water, each with an addition of 0.1% formic acid. The column temperature was set to 35 °C and the injection volume was 5 µL.

#### 3.6.1. Method A

An eluent gradient was applied for the analysis of methanolic extracts and their aqueous and EtOAc phases: 0–10 min: 40%; 10–30 min: 95%; 30–35 min: 80%; 35–40 min: 40% MeOH. Ionization was achieved using ESI in positive and negative scan modes at *m*/*z* 50–2000.

#### 3.6.2. Method B and MS^2^ Measurements

Analysis of the aqueous phases of SBR was performed using an isocratic eluent containing 20% MeOH. MS^2^ experiments were conducted with the aqueous phase of SBR 8 after separation via column chromatography using ions with *m*/*z* at 311, 377, and 463 (negative scan) and 365 and 448 (positive scan). For fragmentation, CID was performed with a normalized collision energy of 35%.

### 3.7. NMR Analysis

NMR measurements were performed on 300 MHz (Bruker AVANCE™ 300 III, Bruker Corporation, Billerica, MA, USA) and 500 MHz (Bruker AVANCE™ 500 NEO, Bruker Corporation, Billerica, MA, USA) devices. Two-dimensional NMR spectroscopy, including 1H,1H-COSY, ^1^H,^13^C-HSQC, and ^1^H,^13^C-HMBC, was used to determine the placement of the signals.

### 3.8. Cell Material

Cell lines Cal-33 and FaDu and human mesenchymal stem cells (hMSCs) were used. Cal-33 cells were obtained from the German Collection of Microorganisms and Cell Cultures (Deutsche Sammlung von Mikroorganismen und Zellkulturen, DMSZ, Braunschweig, Germany) and FaDu cells were obtained from the American Type Cell Collection (ATCC^®^, Manassas, VA, USA) and maintained at 37 °C in a 5% CO_2_ atmosphere in a monolayer in Dulbecco’s modified Eagle’s medium (DMEM) and Ultraglutamine (Lonza, Verviers, Belgium), with 10% fetal calf serum (PAN Biotech GmbH, Aidenbach, Germany) and 1% antibiotic–antimycotic solution (Gibco, Paisley, UK). Confluent cells were passaged via treatment with 0.05% trypsin–0.02% EDTA. hMSCs were isolated from lipoaspirate samples collected from patients undergoing liposuction or lipofilling procedures at the Rostock University Hospital or Plastic Surgery Clinic in Rostock, Germany, with approval from the Ethics Committee at the University Medical Center Rostock No. A 2014-0092. Pure stem cells were kept in stem cell media that contained 45% Iscove’s Modified Dulbecco’s Medium, 45% Gibco^®^ F-12 Nutrient Mixture, and 10% NCS and was supplemented with 0.1 mL Gibco™ Penicillin–Streptomycin (all from Thermo Fisher Scientific GmbH, Regensburg, Germany) and 10 µg of basic fibroblast growth factor (recombinant human bFGF; Millipore Merck KGaA, Darmstadt, Germany) per 1 L of cell culture media.

### 3.9. MTS Assay

Cancer cells were seeded in a 96-well plate with DMEM supplemented with 10% fetal calf serum and 1% antibiotic–antimycotic solution and hMSCs were seeded with Mesenchymal Stem Cell Growth Medium 2 (PromoCell, Heidelberg, Germany). After 24 h of incubation, the medium was changed to phenol-red-free DMEM (Gibco™, Schwerte, Germany; assay medium), after which the cells were incubated for another 24 h. Aqueous and EtOAc phases of the plant extracts were dissolved in DMSO and these solutions were then dissolved in the assay medium, yielding an extract concentration of 50 µg/mL. Cells were treated with the resulting solution and incubated for 24 h. The assay was performed using 10 µL of MTS reagent per well. After 60 min of incubation, the absorbance was measured at 492 nm using an Absorbance 96 plate reader (Byonoy GmbH, Hamburg, Germany) and Absorbance 96 software (Byonoy GmbH, Hamburg, Germany).

### 3.10. Flow Cytometry

Cal-33 and FaDu were seeded in 6-well plates and incubated for 24 h. The cells were washed with phosphate-buffered saline (PBS) solution and the medium was changed to the assay medium. The cells were then incubated for 24 h, washed with PBS, and treated with solutions of the aqueous phase of SBR 8 extract and its fractions 1 and 4 in DMSO, which were dissolved in assay medium to yield a concentration of 50 µg/mL. Cells were incubated for 24 h (Cal-33 and FaDu), 48, or 72 h (only FaDu). The supernatant was transferred to FACS tubes and the cells were washed twice with PBS and trypsinized with 0.05% trypsin-0.02% EDTA for 5 min. The cells were then transferred to FACS tubes using the assay medium. The tubes were centrifuged (5 min, 2000 rpm) and the medium was removed. Cells were fixed with ice-cold 70% ethanol and stored at −20 °C for 48 h. Propidium iodide (PI) staining was performed. Cells were centrifuged, washed with PBS, and incubated with RNAse (1 mg/mL) for 30 min. Then, the RNAse solution was removed. The cell pellet was resuspended in 500 µL of PI solution (50 mg/mL). The cells were left for at least 48 h at 3–8 °C prior to analysis. Flow cytometric measurements were performed using a BD FACSCalibur cytometer (BD Biosciences, San Jose, CA, USA) and CellQuest Pro (BD Biosciences, San Jose, CA, USA). FlowJo software (version 10.6.1, BD Biosciences, San Jose, CA, USA) was used for analysis. A minimum of 10,000 ungated events were recorded. Cell cycle phases G1, G2, S, and Sub G1 were calculated using FlowJo.

### 3.11. ECIS

Electric cell–substrate impedance (ECIS) measurements were conducted using an ECIS^®^ Z-Theta 96 Well Array Station with a 96W20idf PET well plate (Applied BioPhysics, Inc., Troy, NY, USA). ECIS software (version 1.2.186.0 PC, Applied BioPhysics, Inc., Troy, NY, USA) was used to operate the device and analyze the results. FaDu cells were seeded in the well plate and incubated with 200 µL of DMEM until a constant impedance signal was reached (approximately 2 days). Then, 100 µL of the medium was removed from each well except for the cell-free wells and 100 µL of fresh medium was added, in which the aqueous phase of the SBR 8 extract was dissolved, yielding a concentration of 50 µg/mL. As for the control, the cells were also treated with DMEM, as well as DMEM with 2 µL of DMSO. Their impedance (Z), capacity (C), and resistance (R) were measured at 9 frequencies (250, 500, 1000, 2000, 4000, 8000, 16,000, 32,000, and 64,000 Hz) over a period of 73 h. For the recovery experiment, the medium in the wells was removed completely and replaced with 200 µL of fresh medium. Measurements were carried out for an additional 48 h. Data were analyzed using ECIS software. A frequency analysis was performed and 16,000 Hz was chosen to as the frequency the impedance results would be viewed at.

### 3.12. Live/Dead Staining

Cal-33 and FaDu cells were treated with DMSO (control) and SBR 8 for 24, 48, and 72 h prior to the fluorescence labeling of living (green) and dead (red) cells using the two-color fluorescent probes Calcein-AM and EthD-III provided by PromoKine (PK-CA707-30002, Heidelberg, Germany). Notably, living cells convert non-fluorescent cell-permanent Calcein-AM to intensely green-fluorescent Calcein by ubiquitous intracellular esterase activity. The red Ethidium Homodimer III (EthD-III) dye enters cells with damaged membranes and undergoes an enhancement of its fluorescence upon binding nucleic acids.

## 4. Conclusions

In our study we analyzed the effect of SBR on tumorigenic and non-tumorigenic cells using a metabolic activity assay, flow cytometry, and ECIS. We observed a viability inhibition in FaDu and Cal-33 treated with the aqueous phase of SBR 8. In non-tumorigenic cells we did not see an inhibition. On the contrary, SBR 8 had a positive effect on hMSC viability. This indicates that the extracts might be powerful agents in combinatory chemotherapy, potentially taking over part of the treatment of cancer cells, while not harming and possibly even protecting non-tumorigenic cells. We separated the extract into fractions and were able to determine two fractions (fraction 1 and 4) which we were able to correlate with the bioactive effects of the extract using a metabolic activity assay. To validate these results, we performed flow cytometry (Cal33 and FaDu) and ECIS (only FaDu) experiments. The results indicate a cell cycle arrest in the G2 phase and possible subsequent apoptosis, which began after approximately 48 h of treatment.

We were able to analyze the fractions using LC/MS^2^. They seemed to contain at least two substances which could be correlated with the observed bioactive effects. We propose that SBR 8 contains a 7-(2-hydroxyethyl)-8-methoxy-2′-hydroxyflavone, which we suspect to be at least partially responsible for the observed bioactivity.

Further studies of SBR 8 are crucial to identify the other compounds it contains, especially within fractions 1 and 4. Additionally, the effect of 7-(2-hydroxyethyl)-8-methoxy-2′-hydroxyflavone on cells has to be investigated. However, in this study SBR extracts proved to be of interest for cancer chemotherapy.

## Data Availability

The data that support the findings of this study are available from the corresponding author, Nadja Engel, upon reasonable request.

## Supplementary Materials

The following supporting information can be downloaded at https://www.mdpi.com/article/10.3390/ijms26104625/s1.

## Abbreviations

The following abbreviations are used in this manuscript:

LC	Liquid chromatography
MS	Mass spectrometry
ESI	Electrospray ionization
CID	Collision-induced dissociation
SBR	Sea buckthorn root
DMSO	Dimethyl sulfoxide
DMEM	Dulbecco’s modified Eagle’s medium
hMSCs	Human mesenchymal stem cells
EDTA	Ethylenediaminetetraacetic acid
NCS	Newborn calf serum
ECIS	Electric cell–substrate impedance sensing
PBS	Phosphate-buffered saline
Rpm	Rounds per minute
PI	Propidium iodide
PTX	Paclitaxel
