# Supplementary material for: Anti-Tumorigenic Effects of Sea Buckthorn Root Extracts on Head and Neck Cancer Cells—A Systematic Analysis
Source: Int J Mol Sci. 2025 May 12;26(10):4625. doi: 10.3390/ijms26104625 (PMC12110798; doi:10.3390/ijms26104625)
Supplement: Supplementary file 1 [file ijms-26-04625-s001.zip › ijms-3525896-supplementary.pdf]

Supporting Information

Anti-Tumorigenic Effects of Sea Buckthorn Root Extracts on Head and Neck Cancer Cells—A Systematic Analysis

Alina Gazizova 1, Manuel Gronbach 1, Christina Oppermann 1, Udo Kragl 1,2 and Nadja Engel 2,3,*

[1]Department of Industrial and Analytical Chemistry, Institute of Chemistry, University of Rostock, Albert-Einstein-Straße 3a, 18059 Rostock, Germany
[2] Department of Life, Light and Matter, University of Rostock, Albert-Einstein-Straße 25, 18059 Rostock, Germany

[3]Department of Oral, Maxillofacial and Plastic Surgery, University Medicine Rostock, Schillingallee 35, 18057 Rostock, Germany
** Correspondence: nadja.engel@med.uni-rostock.de; Tel.: +49-381-494-6679*

1. Growing Conditions

Supplementary Table S1 Growing Conditions of the 8 sea buckthorn roots.

Root No.	Soil
1	Field sand
2	80% field sand, 20% plant soil
3	80% field sand, 20% playing sand
4	60% field sand, 20% plant soil, 20% playing sand
5	Field sand, in a big pot
6	60% field sand, 20% plant soil, 20% playing sand, in a big pot
7	50% field sand, 50% plant soil, in a big pot
8	100% plant soil, in a big pot

2. Comparison of classical (reflux) and microwave extracts of sea buckthorn roots

The liquid chromatograms of the roots are given in the following Supplementary Figures.

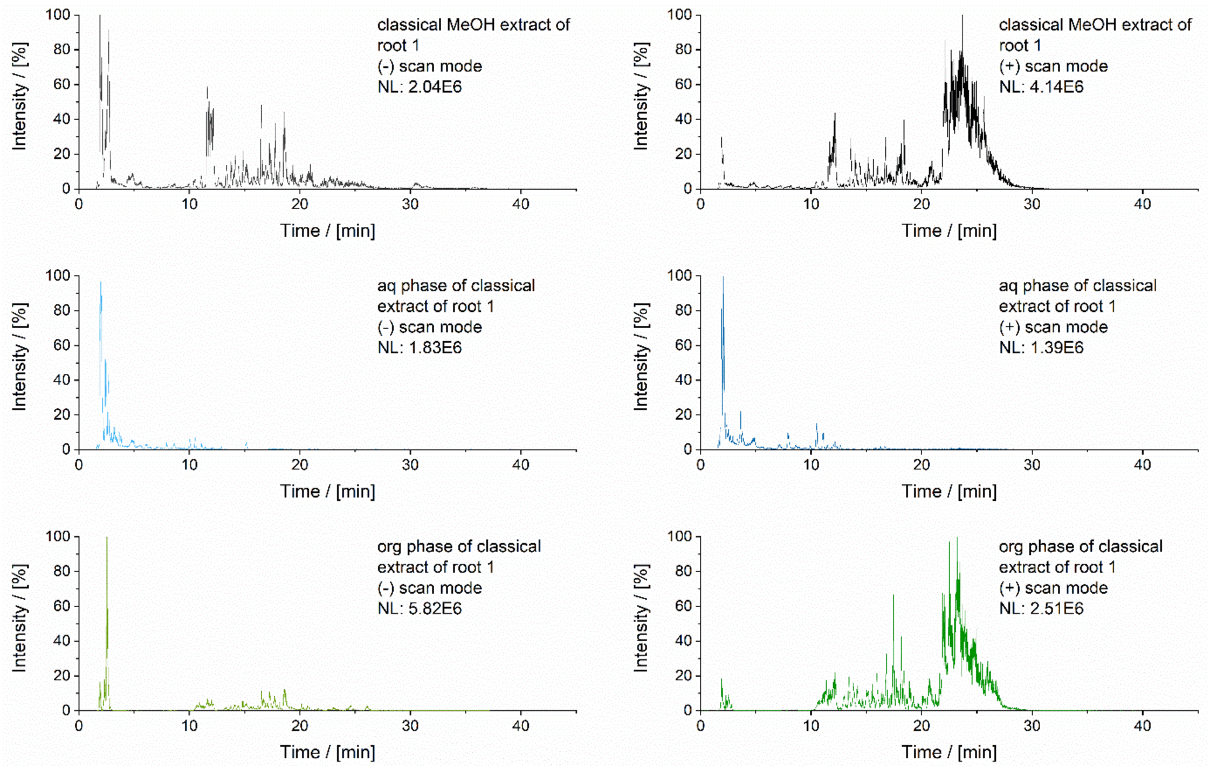

Supplementary Figure S1 Base peak chromatogram of classical extract of root 1.

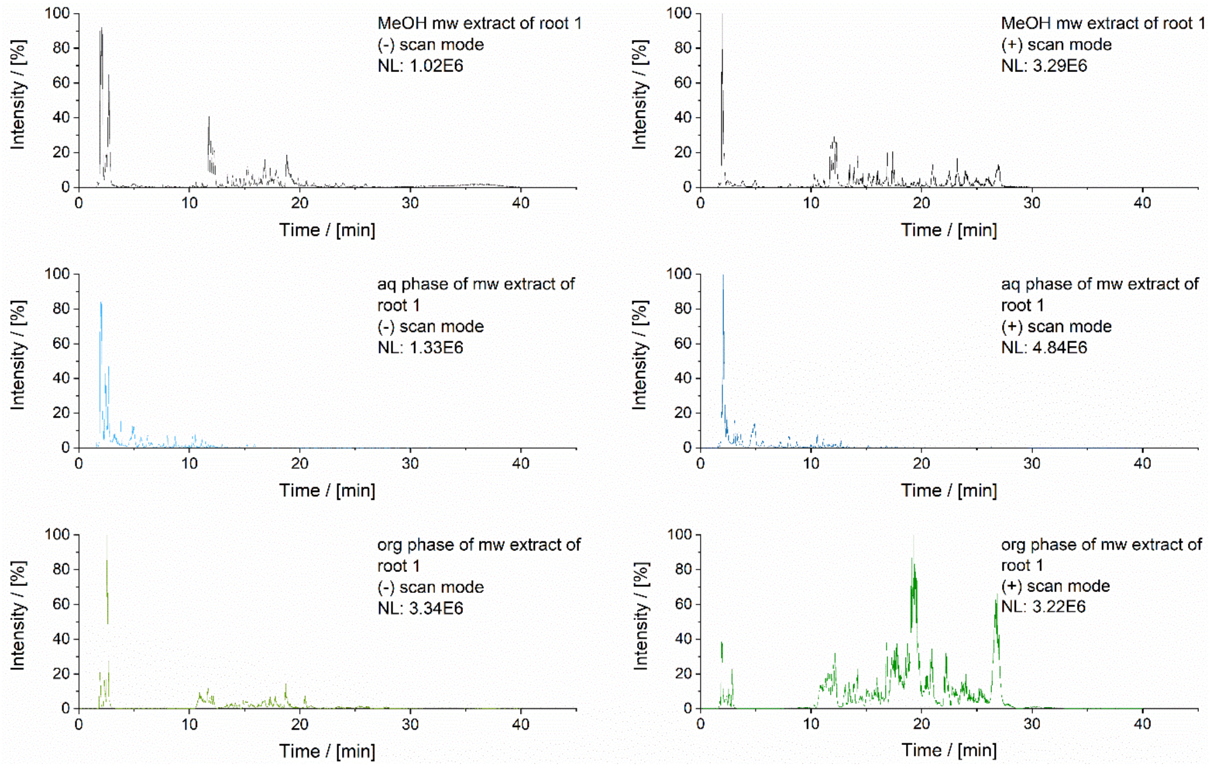

Supplementary Figure S2 Base peak chromatogram of microwave extract of root 1.

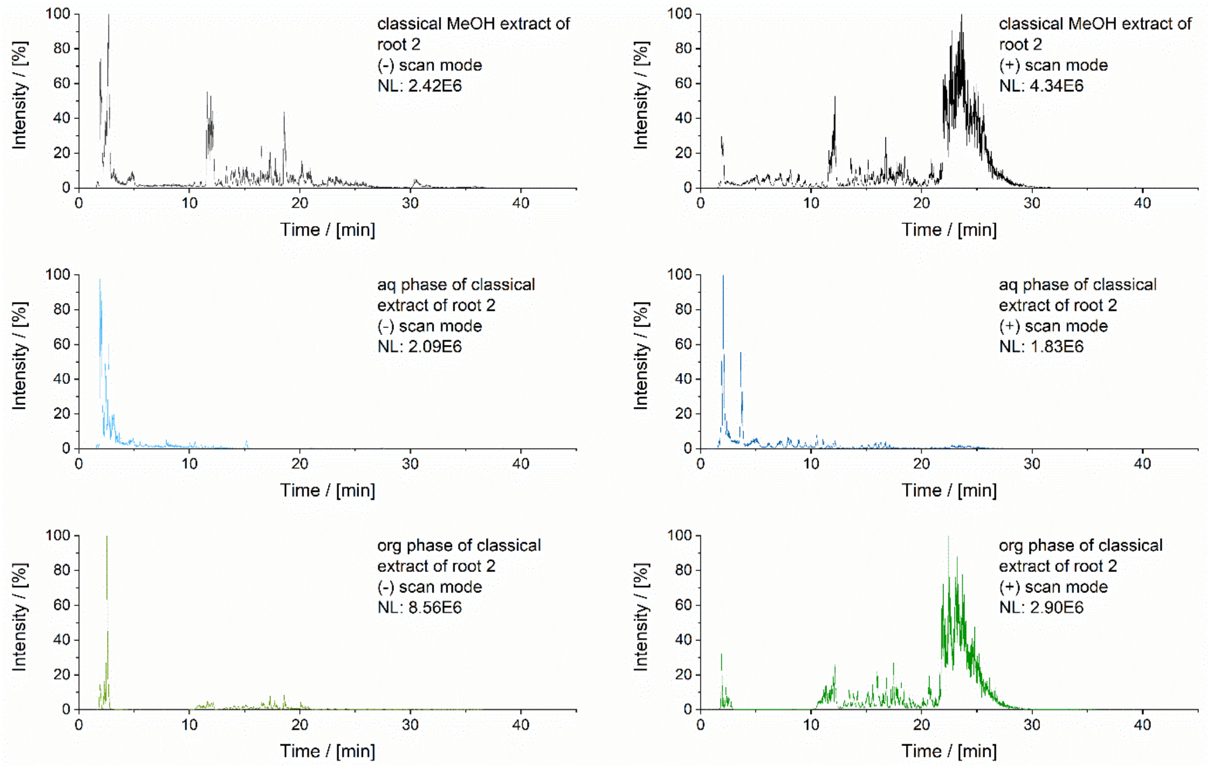

Supplementary Figure S3 Base peak chromatogram of classical extract of root 2.

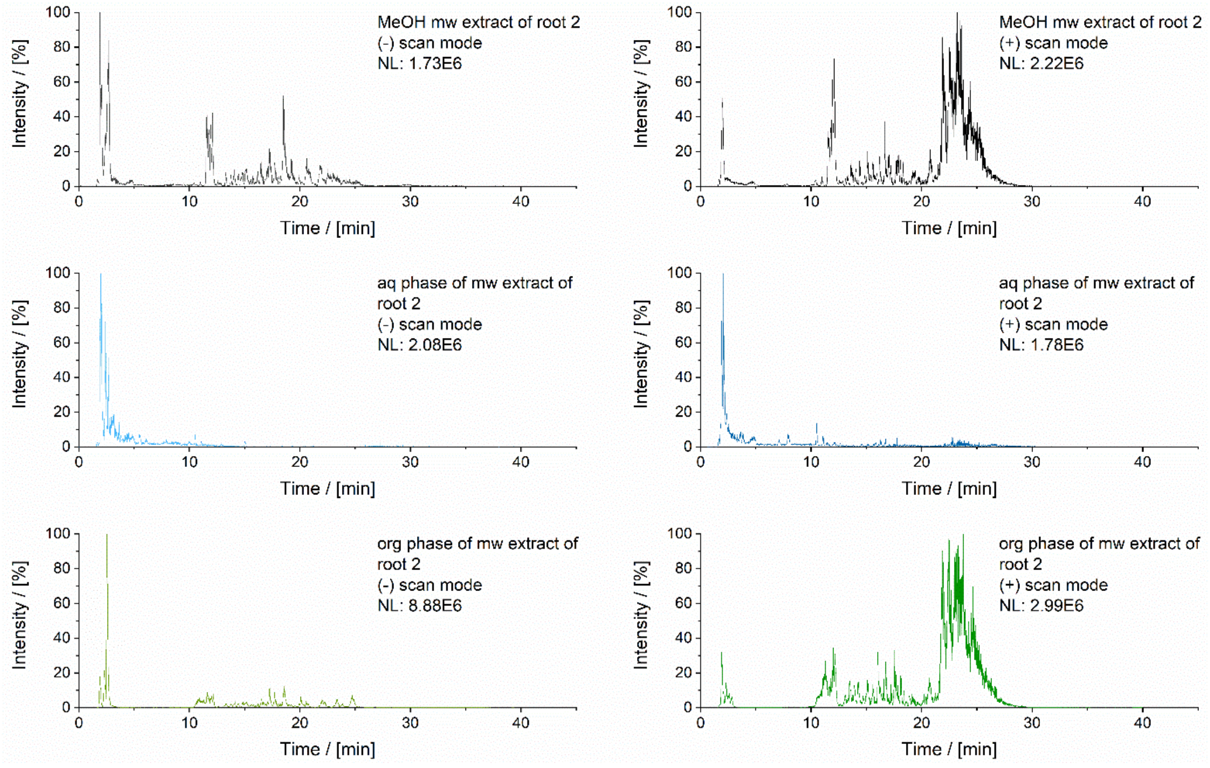

Supplementary Figure S4 Base peak chromatogram of microwave extract of root 2.

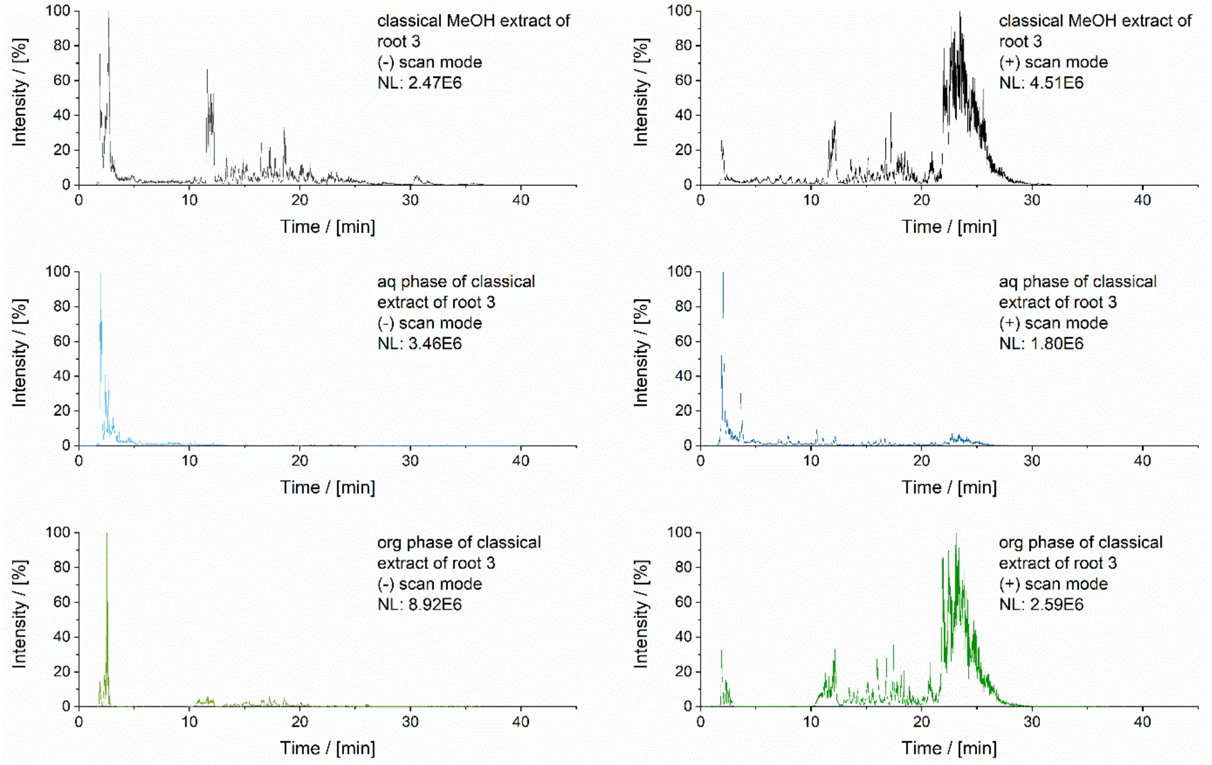

Supplementary Figure S5 Base peak chromatogram of classical extract of root 3.

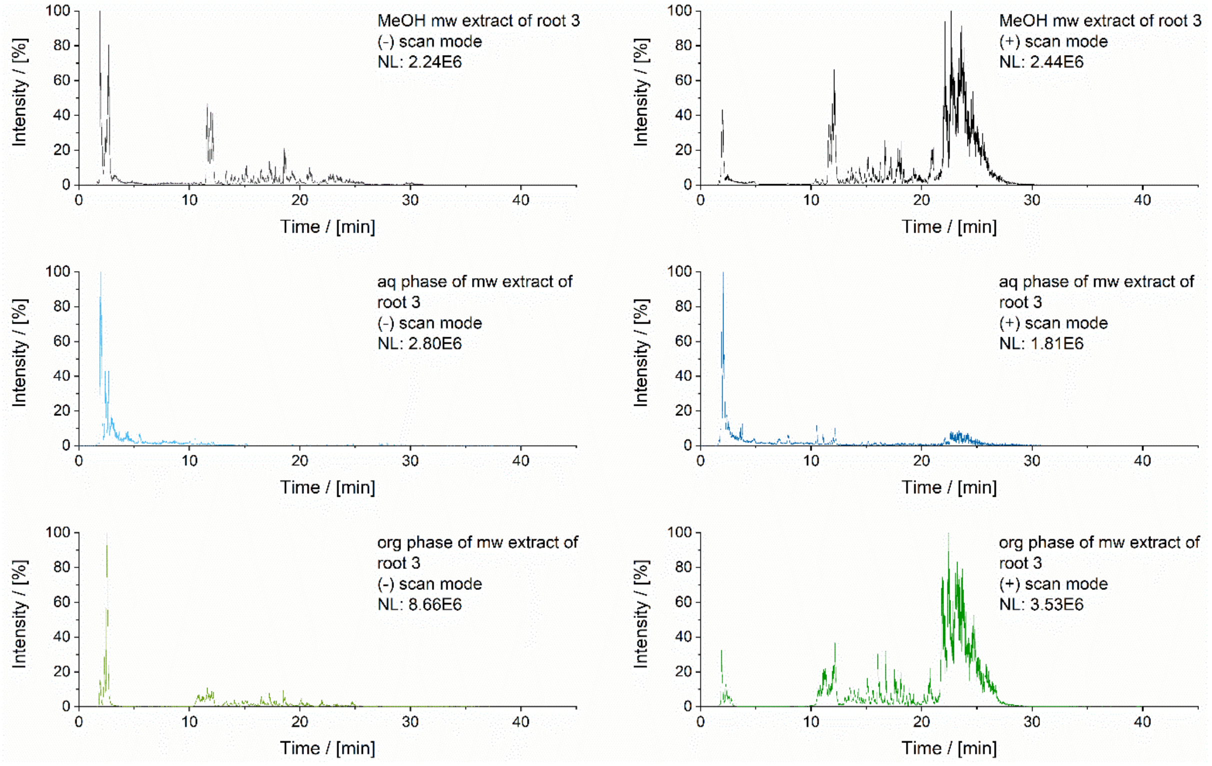

Supplementary Figure S6 Base peak chromatogram of microwave extract of root 3.

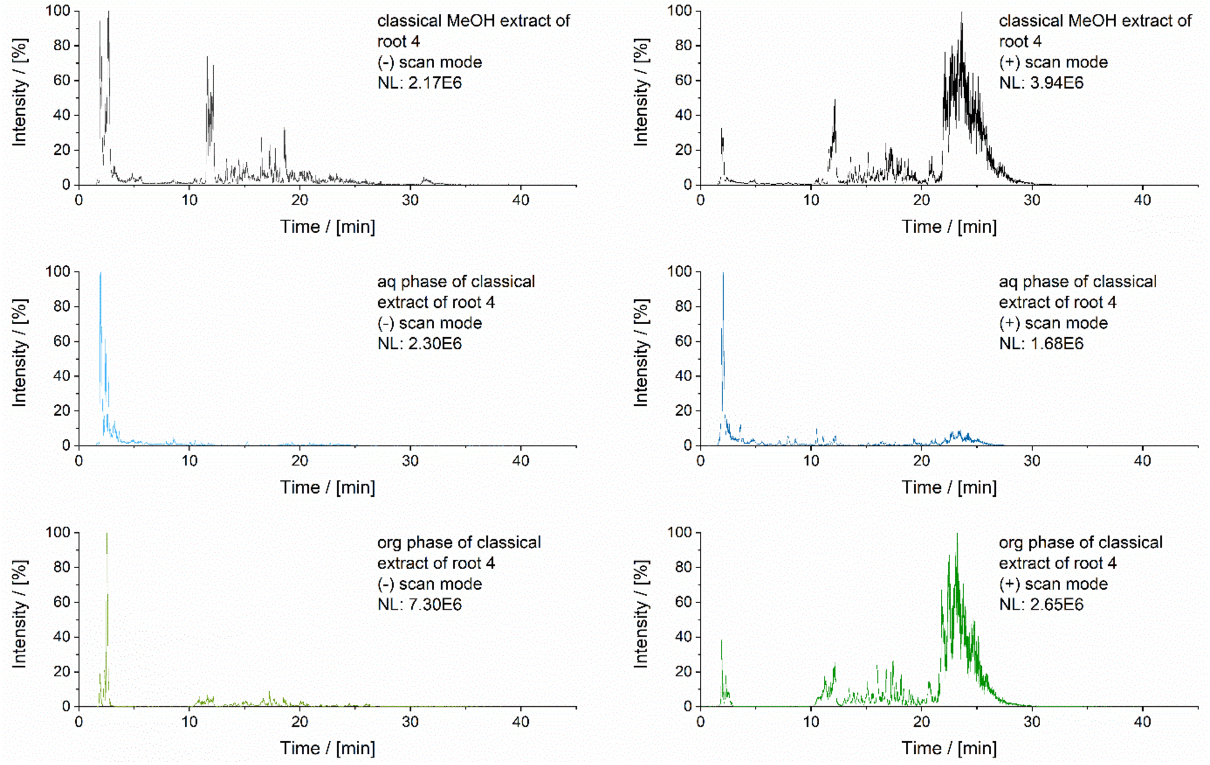

Supplementary Figure S7 Base peak chromatogram of classical extract of root 4.

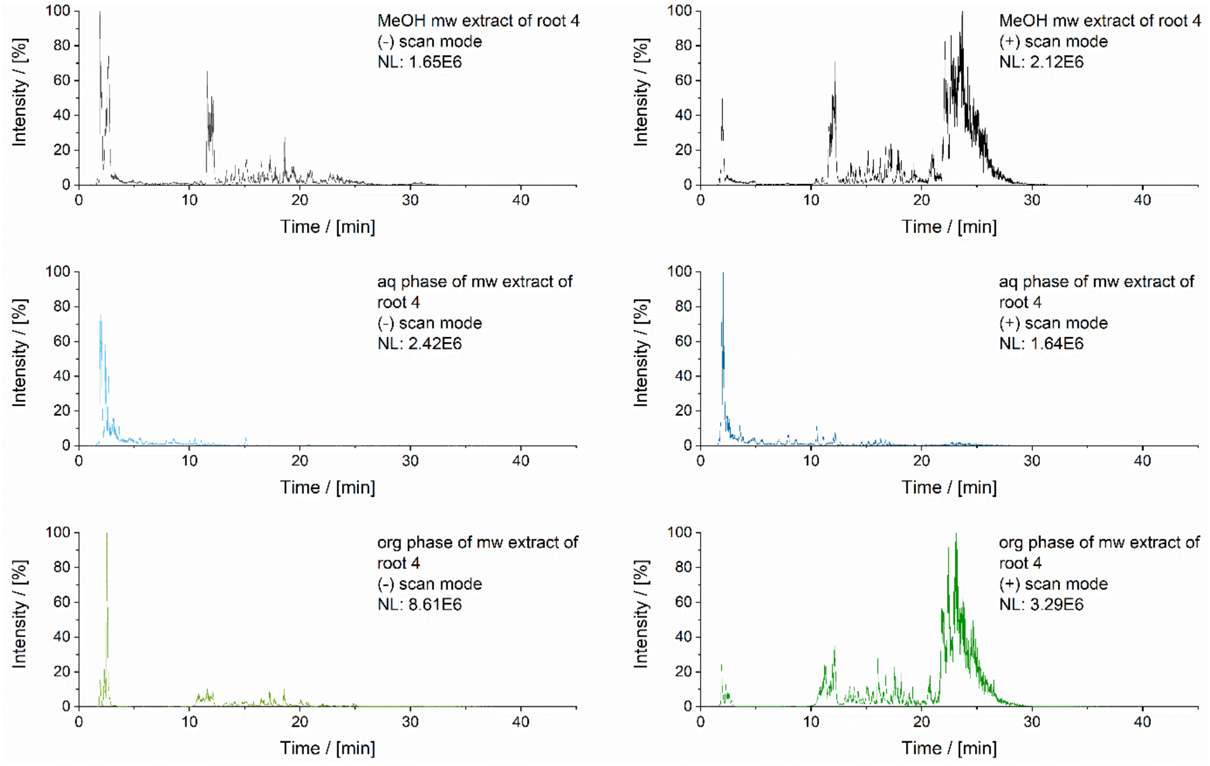

Supplementary Figure S8 Base peak chromatogram of microwave extract of root 4.

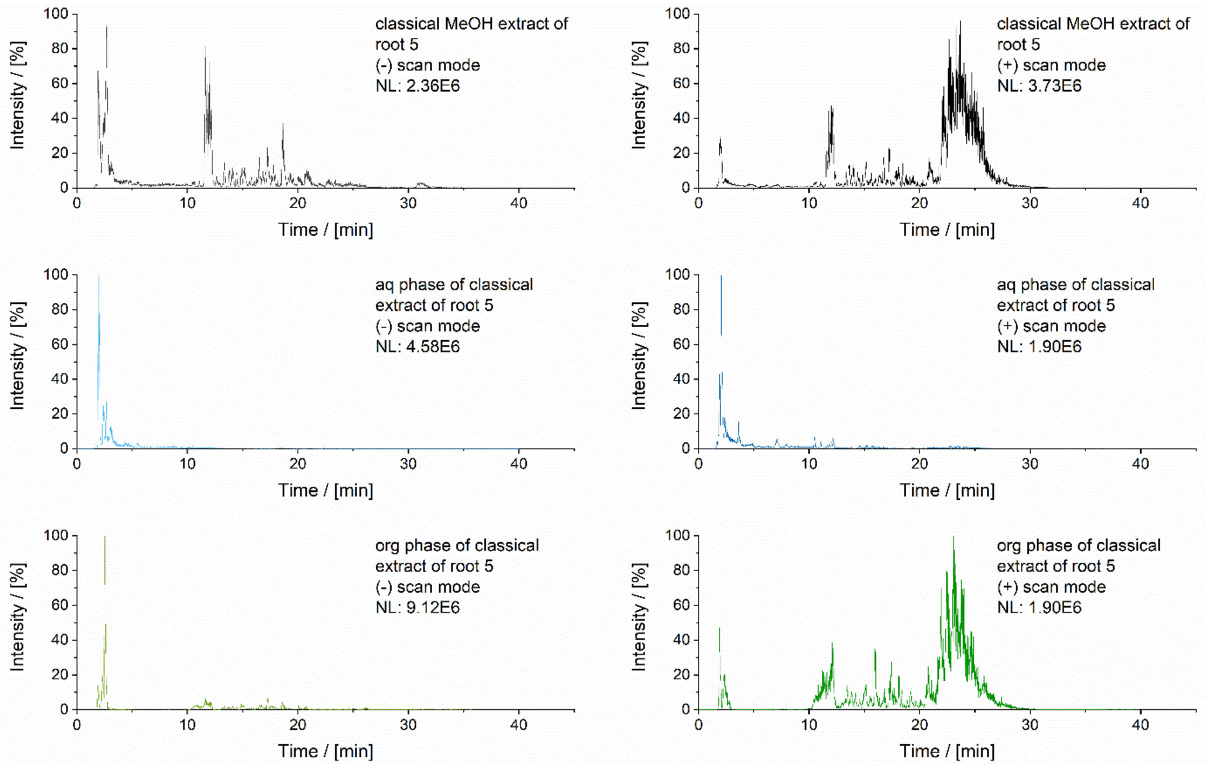

Supplementary Figure S9 Base peak chromatogram of classical extract of root 5.

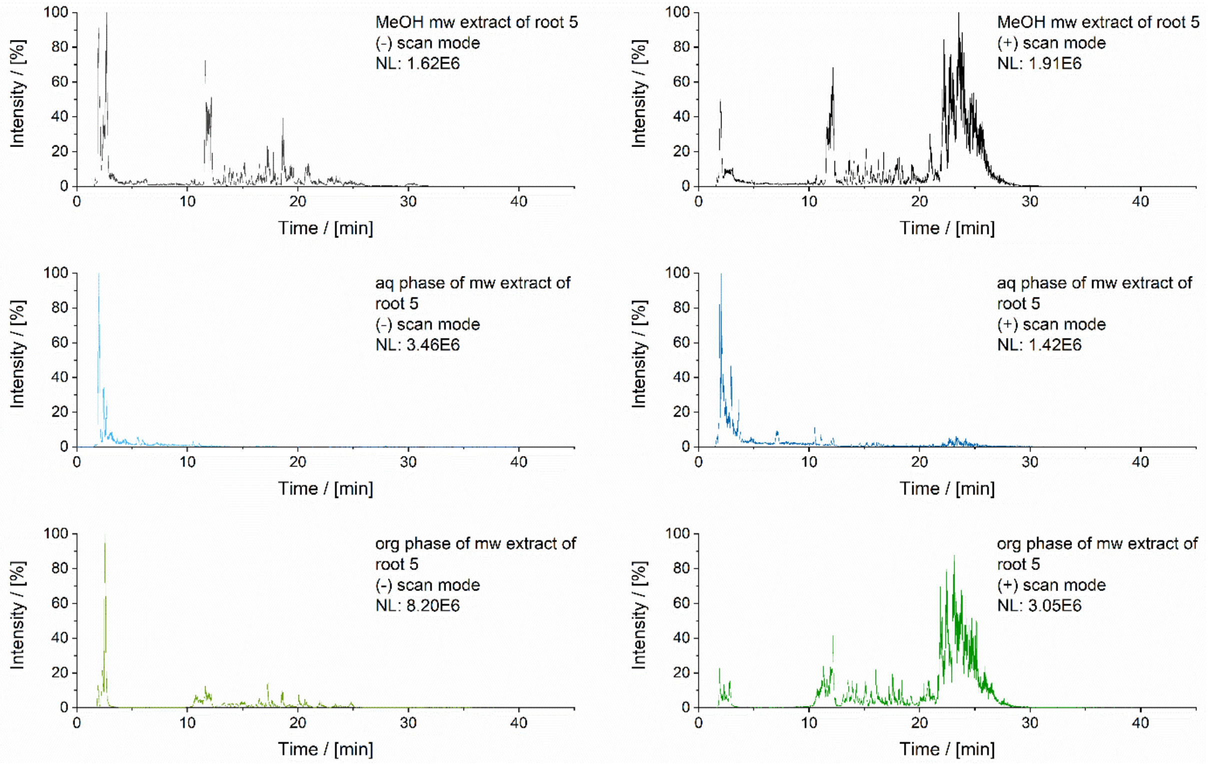

Supplementary Figure S10 Base peak chromatogram of microwave extract of root 5.

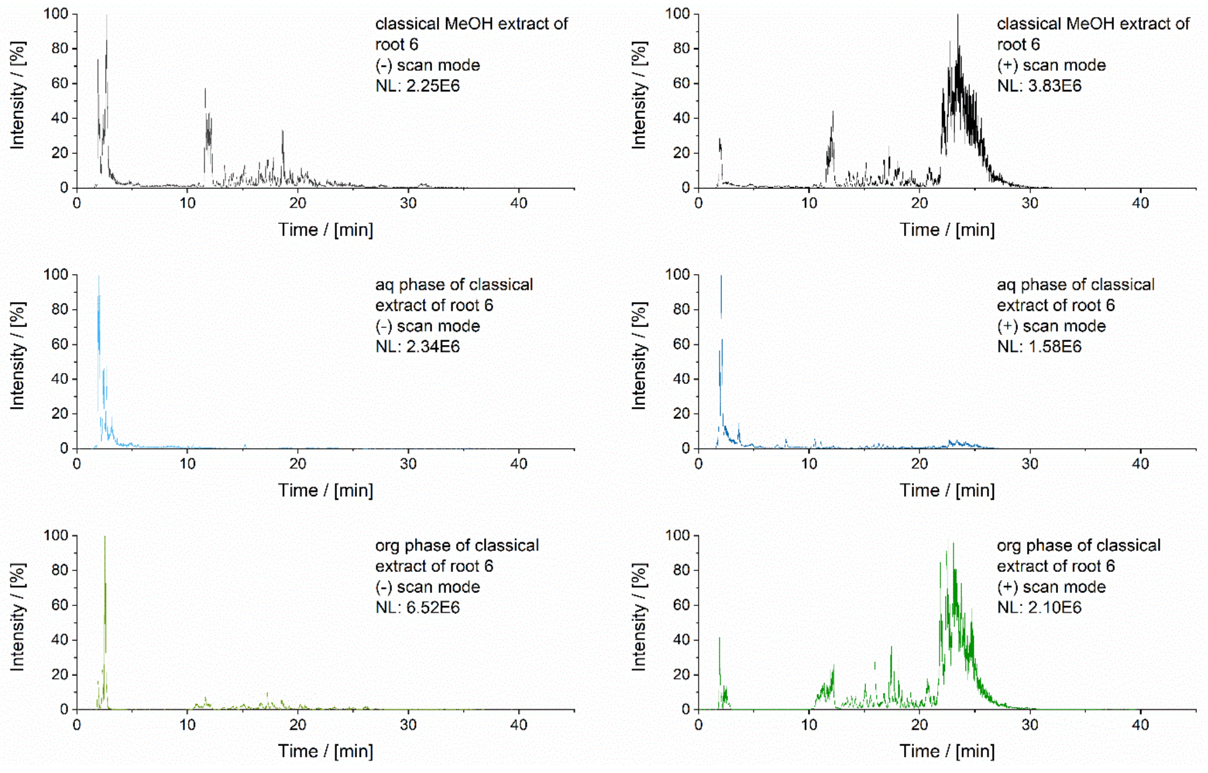

Supplementary Figure S11 Base peak chromatogram of classical extract of root 6.

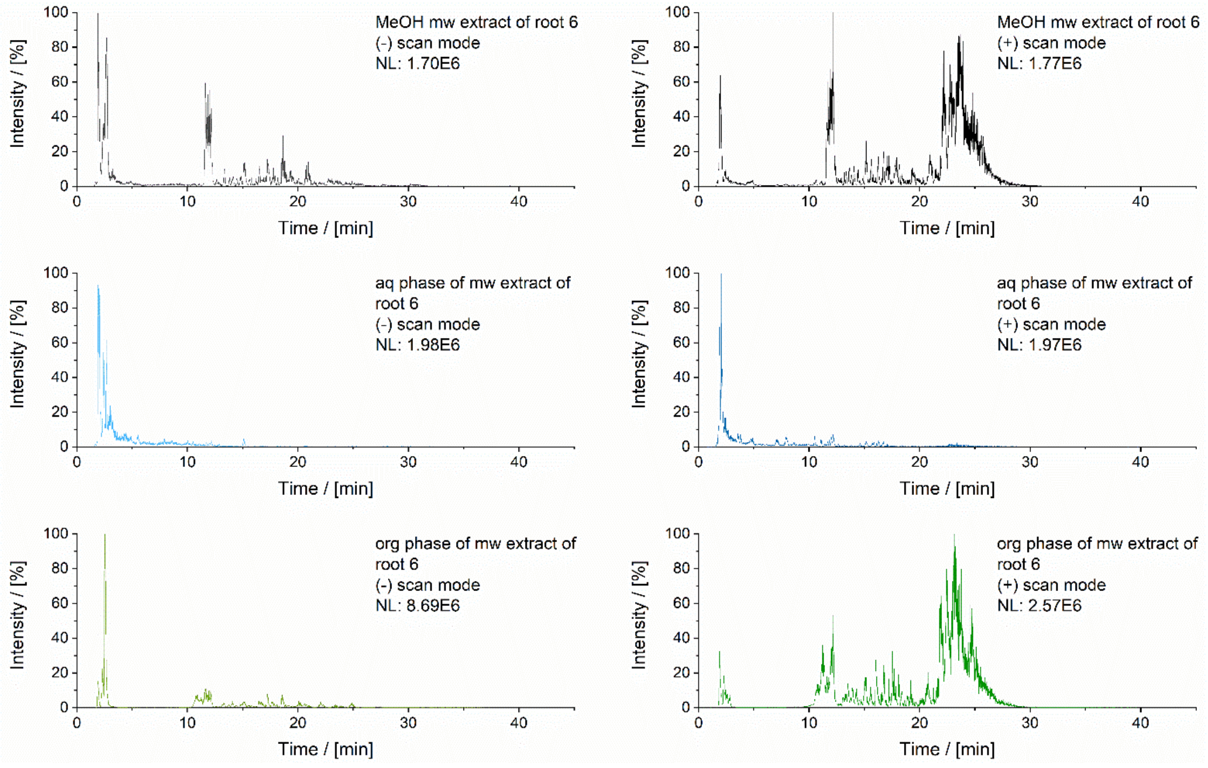

Supplementary Figure S12 Base peak chromatogram of microwave extract of root 6.

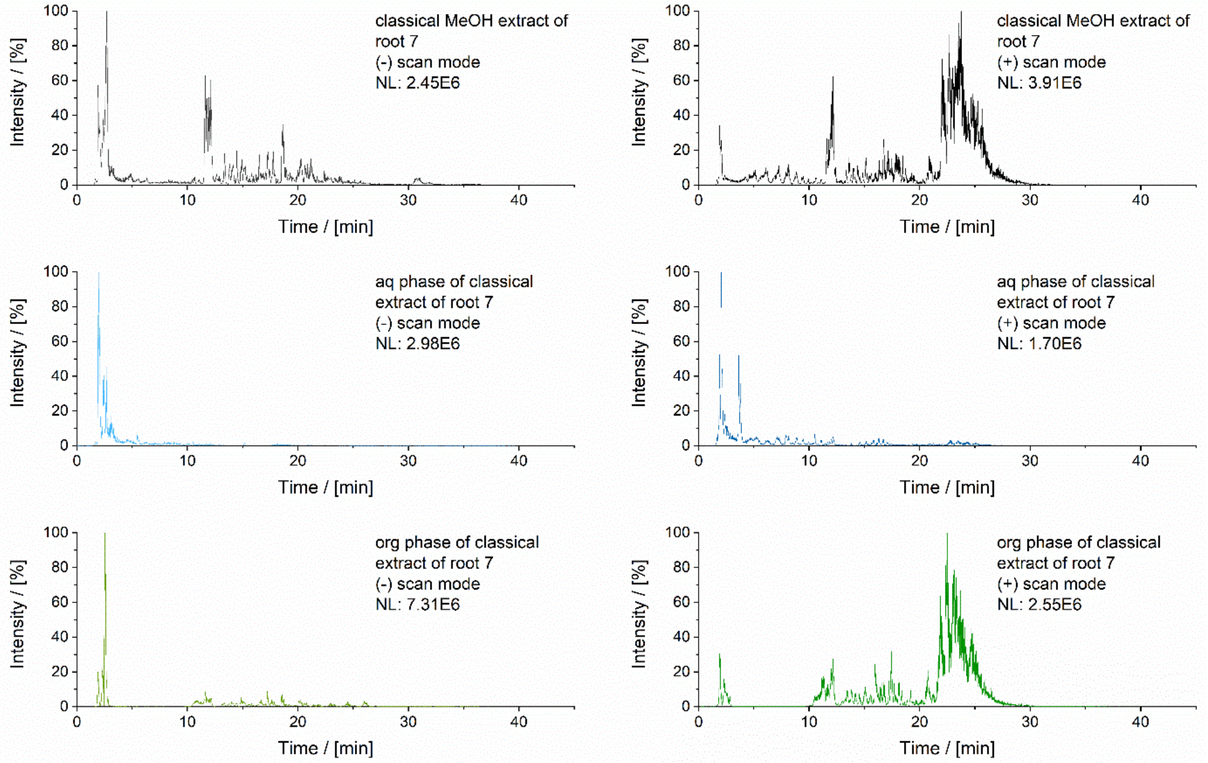

Supplementary Figure S13 Base peak chromatogram of classical extract of root 7.

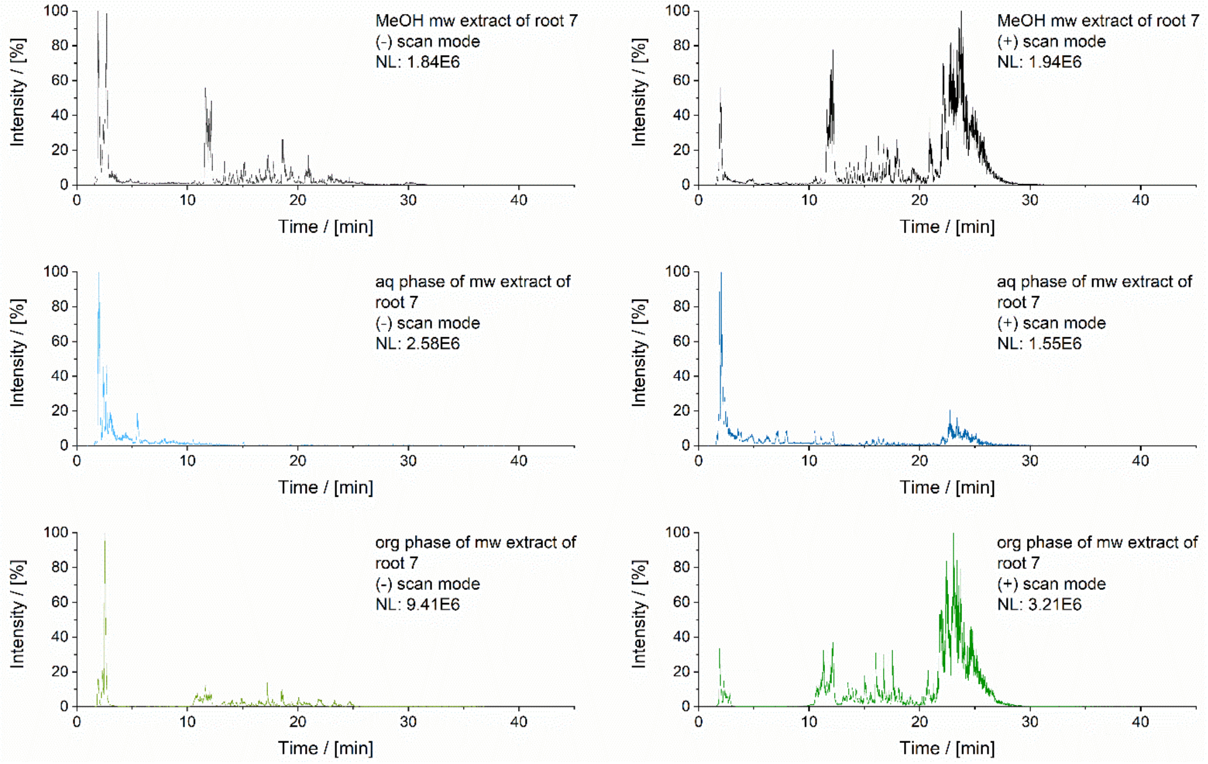

Supplementary Figure S14 Base peak chromatogram of microwave extract of root 7.

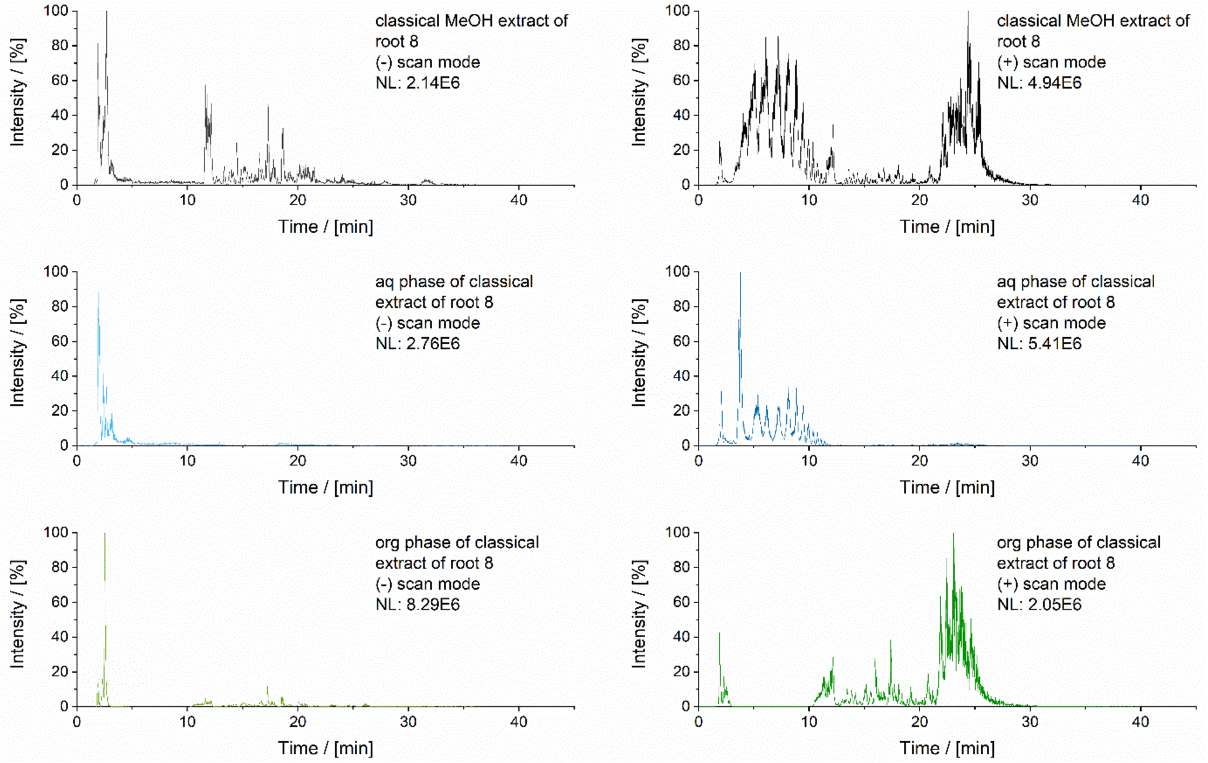

Supplementary Figure S15 Base peak chromatogram of classical extract of root 8.

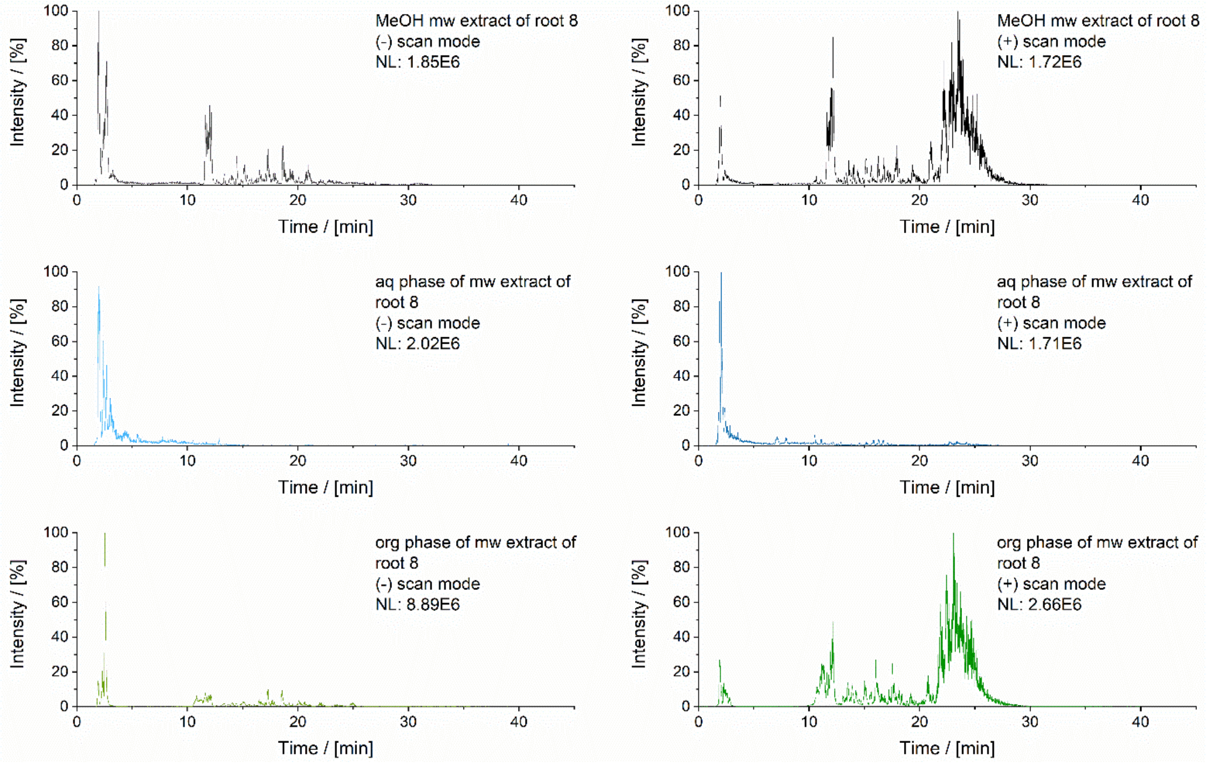

Supplementary Figure S16 Base peak chromatogram of microwave extract of root 8.

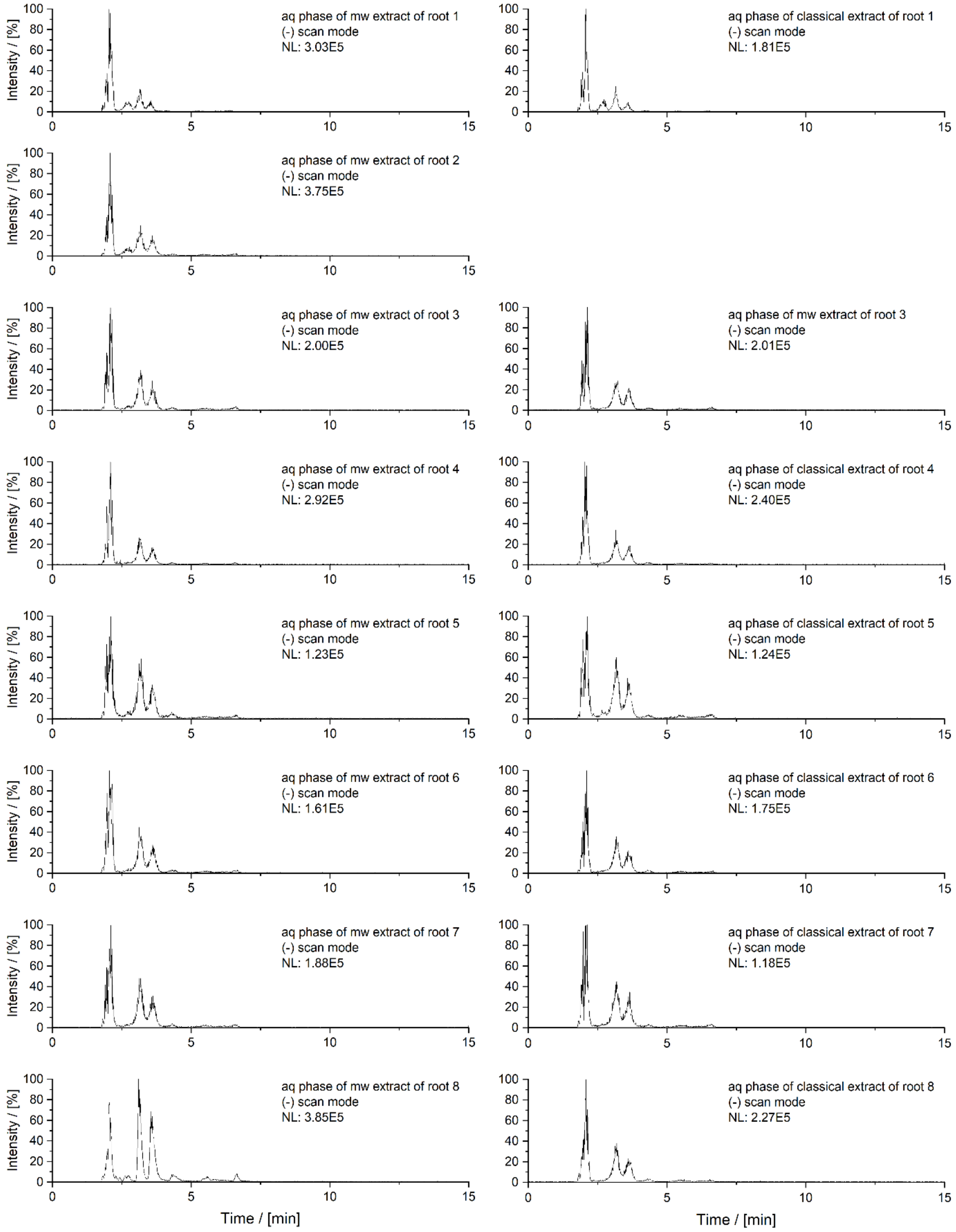

Supplementary Figure S17 Base peak chromatograms of microwave (left) and classical (right) extracts of roots 1 – 8 in (-) scan mode.

Please note that a sample of the aqueous phase of the classical MeOH extract of sea buckthorn root 2 was not available anymore and therefore no measurement can be displayed.

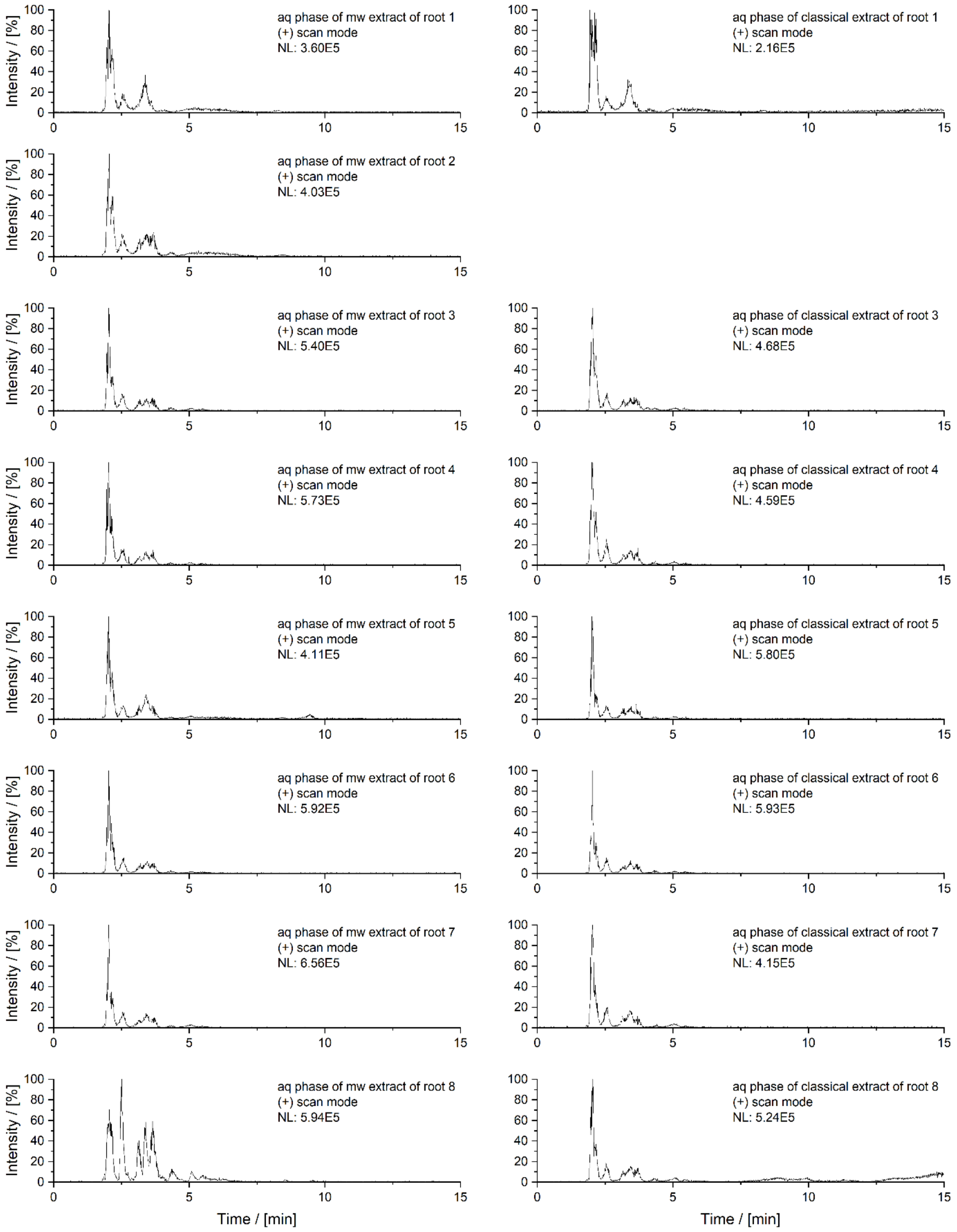

Supplementary Figure S18 Base peak chromatograms of microwave (left) and classical (right) extracts of roots 1 – 8 in (+) scan mode.

Please note that a sample the aqueous phase of the classical MeOH extract of sea buckthorn root 2 was not available anymore and therefore no measurement can be displayed.

Supplementary Table S2 Retention times and m/z of common peaks between the microwave extract of root 8, its aqueous phase and the aqueous phase's fraction 1.

t [min]	m/z fraction 1	m/z	m/z

			Aqueous phase of MeOH microwave extract	MeOH microwave extract
Negative scan mode				
	1.69	289 (37), 273 (55), 227 (84), 159 (100)	401 (33), 375 (100), 349 (68), 159 (59)	275 (31), 274 (31), 273 (100), 289 (30) 159 (54)
	2.36	578 (32), 577 (100), 163 (35), 161 (46)	866 (48), 865 (100), 577 (81)	866 (35), 865 (63), 578 (35), 577 (100), 289 (80)
	2.58	875 (30), 289 (100)	1443 (32), 1442 (58), 1441 (68),	289 (100)
Positive scan mode				
	1.91	431 (38), 226 (100)	381 (100)	1041 (31), 754 (34), 753 (97), 465 (50), 381 (35), 160 (100), 156 (34), 133 (31)

Supplementary Table S3 Retention times and m/z of common peaks between the microwave extract of root 8, its aqueous phase and the aqueous phase's fraction 4.

	t [min]	m/z fraction 4	m/z Aqueous phase of MeOH microwave extract	m/z MeOH microwave extract
Negative scan mode				
	1.93	341 (100)	341 (89), 131 (100)	341 (74), 191 (58), 131 (100)
	2.07	379 (34), 377 (100)	377 (66), 191 (100)	439 (30), 377 (70), 341 (55), 191 (100)
Positive scan mode				
	1.95	465 (37), 448 (100), 296 (32), 289 (52), 160 (43)	381 (100), 365 (69)	1041 (30), 754 (33), 753 (90), 465 (53), 448 (37), 289 (31), 160 (100), 159 (31)
	2.23	753 (46), 465 (100), 448 (62), 289 (41)	1907 (47), 1906 (63), 1905 (65), 1619 (41), 1618 (63), 1617 (80), 1331 (37), 1330 (68), 1329 (100), 1042 (62), 1041 (85), 753 (70), 577 (37), 465 (80), 448 (41), 203 (39), 174 (32), 172 (37), 160 (88), 159 (96)	1908 (35), 1618 (34), 1617 (42), 1330 (56), 1329 (76), 1042 (39), 1041 (60), 579 (38), 578 (38), 577 (57), 465 (45), 409 (35), 365 (61), 289 (100), 174 (44), 160 (82), 159 (63), 139 (34), 123 (58), 115 (36)
	2.35	753 (57), 465 (100), 448 (53), 289 (55)	1027 (42), 868 (47), 867 (89), 579 (41), 577 (43), 289 (37), 161 (48), 160 (30), 144 (100), 143, 117 (33), 114 (44)	890 (45), 889 (100). 867 (41), 601 (58), 579 (44), 577 (37), 409 (32), 365 (67), 289 (36), 247 (39), 160 (37), 159 (30), 144 (51)

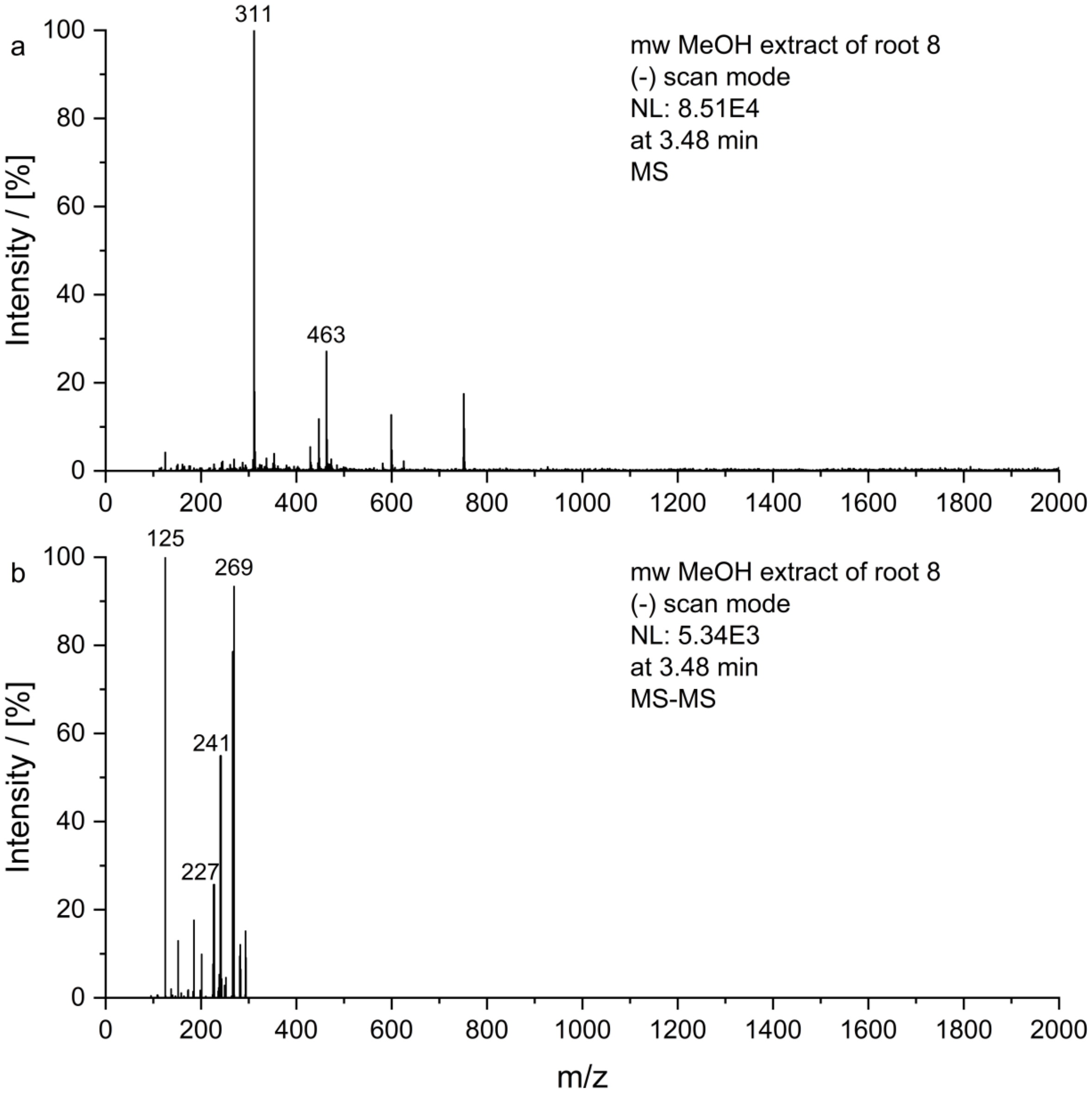

Supplementary Figure S19 Mass spectrum of the signal at 3.48 min in the negative scan mode base peak chromatogram of aqueous phase the MeOH microwave extract of root 8 after ionization (a) and further ionization of the peak with m/z 311 (b).

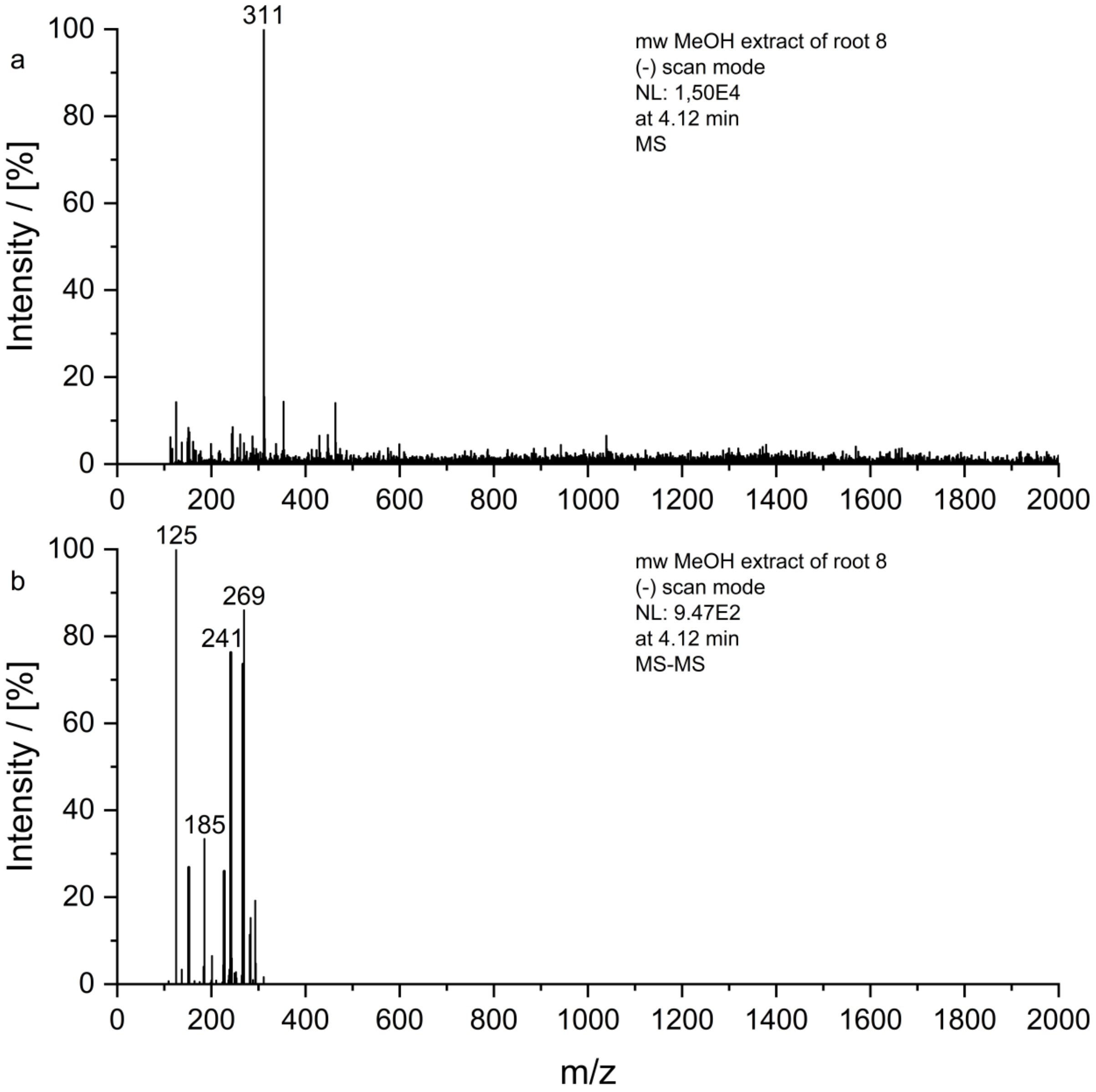

Supplementary Figure S20 Mass Spectrum of the signal at 4.12 min in the negative scan mode base peak chromatogram of aqueous phase the MeOH microwave extract of root 8 after ionization (a) and further ionization of the peak with m/z 311 (b).

3. TLC solutions

Anis aldehyde solution

To create a dipping solution, 0.5 ml anis aldehyde were dissolved in concentrated acetic acid and 1 ml of concentrated sulfuric acid was added. The solution was dissolved in 300 ml ethanol.

4. Sucrose and XRD analysis

After a separation of the microwave extract of root 8 via column chromatography, sucrose was isolated and characterized as sucrose using NMR and XRD. For single crystal X-ray measurements, a diffractometer (Apex D8 Quest, Bruker Corporation, Billerica, USA) with Mo-Kα radiation of the wavelength λ = 0,71073 Å was used. The measurements were taken at -150 °C, which was achieved using a 700 series cryosystem cooler (Oxford Cryosystems, Oxford) with liquid nitrogen. The resulting structure is shown in Supplementary Figure S21 and is identified as sucrose. The NMR spectrum of this substance is given in Supplementary Figure S22.

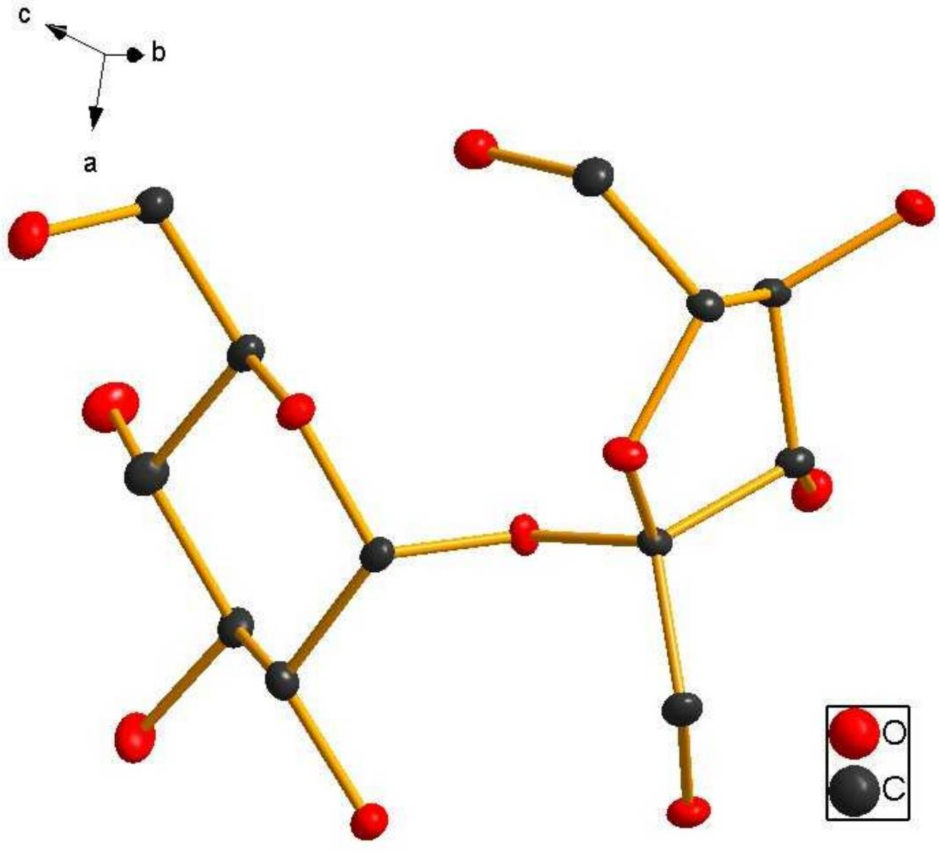

Supplementary Figure S21 Structure determined using X-ray crystallography of crystals.

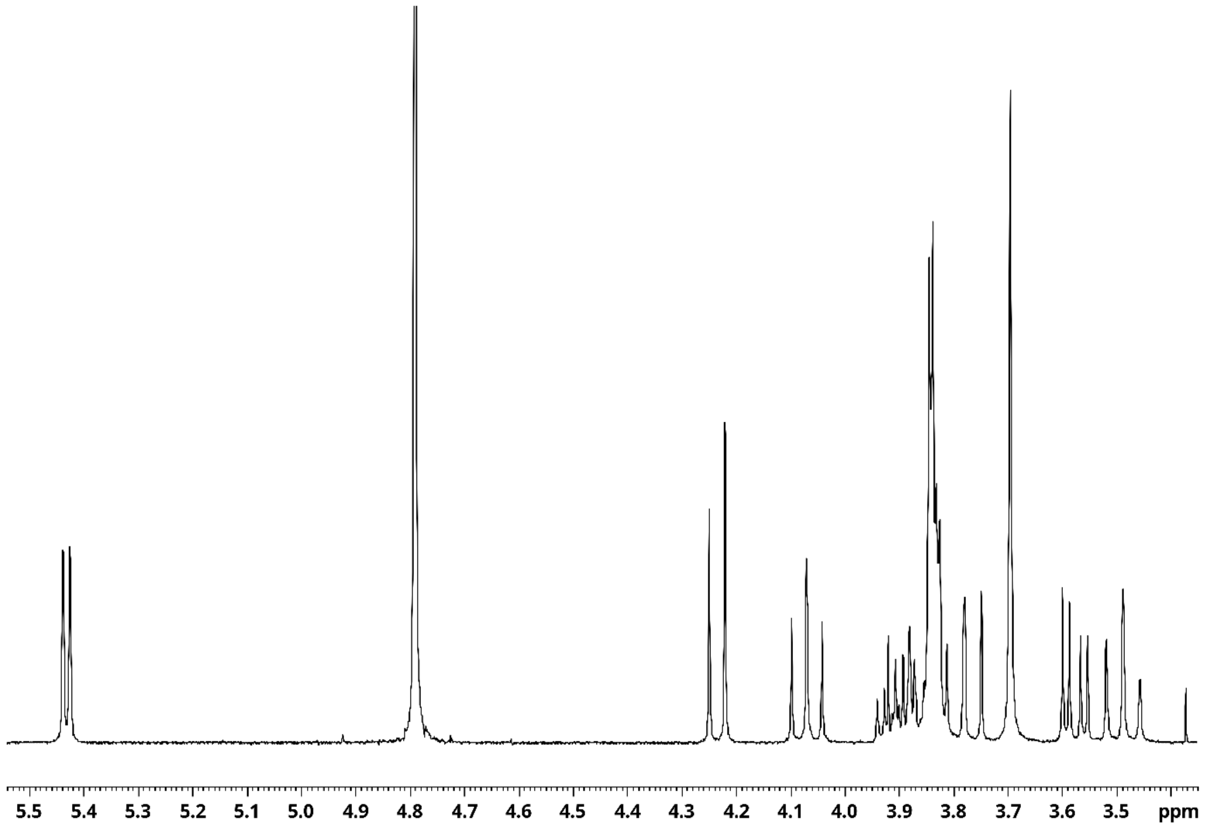

Supplementary Figure S22 NMR spectrum of sucrose isolated from MeOH extract of sea buckthorn root 8 in D2O.

Within the negative scan mode of the ESI-MS spectrum sucrose displays peaks at m/z 387 and 341, which are caused by an $[M + HCOO]^-$ adduct and $[M\text{-}H]^-$ ion respectively. Those signals are also present in the LC-MS spectra of the aqueous phase of the microwave extract of root 8, as well as fraction 1 and 4, which caused the changes in the cell cycle of Cal-33 and FaDu.

5. Catechin

The following Supplementary Figure shows the NMR spectrum of catechin isolated from the ethyl acetate phase of root 4. Acetone-d6 was used as a solvent.

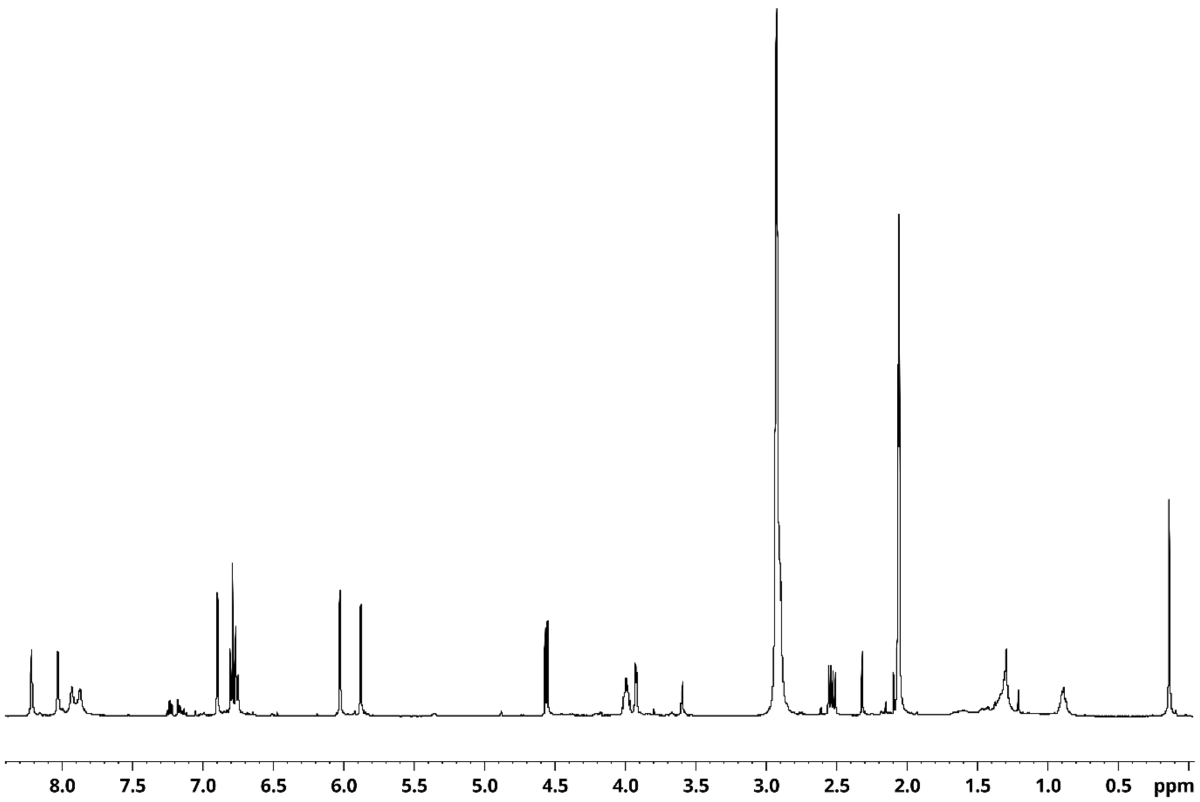

Supplementary Figure S23 NMR spectrum of catechin isolated from ethyl acetate phase of root 4 in acetone-d6.

6. MTS assays

The results of the MTS assays are given in the following Supplementary Tables. Values marked in grey were regarded as outliners and excluded from analysis.

Supplementary Table S4 Viability of FaDu after 24 h treatment with aqueous phases of classical extracts of sea buckthorn roots 1 ¬ 8.

	DMSO	Root 1	Root 2	Root 3	Root 4	Root 5	Root 6	Root 7	Root 8
A	0,3003	0,6792	0,4853	0,4554	0,4145	0,4348	0,4819	0,4675	0,4218
B	0,6163	0,7209	0,687	0,6289	0,7912	0,6294	0,6875	0,6351	0,509
C	0,5562	0,8248	0,7515	0,7228	0,7738	0,6434	0,7167	0,6636	0,5469
D	0,6221	0,8529	0,7501	0,6532	0,7774	0,642	0,8663	0,7051	0,6174
E	0,5312	0,8298	0,7972	0,6705	0,7984	0,6494	0,7325	0,7183	0,6369
F	0,6138	0,8312	0,7758	0,651	0,8434	0,6432	1,1283	0,7142	0,7006
G	0,7138	0,7615	0,6503	0,5664	0,712	0,5665	0,6643	0,6438	0,5698
H	0,2163	0,2975	0,2653	0,3529	0,285	0,4657	0,3804	0,534	0,2709
mean	0,609	0,804	0,735	0,649	0,783	0,629	0,799	0,680	0,597
Standard deviation	0,063	0,051	0,056	0,051	0,043	0,031	0,176	0,037	0,069
t-value		0,000	0,004	0,259	0,000	0,508	0,045	0,045	0,757
Mean [%]	100,00	131,96	120,76	106,55	128,54	103,30	131,26	111,68	98,01
Standard deviation [%]	10,41	6,33	7,57	7,91	5,45	4,98	22,01	5,45	11,55

Supplementary Table S5 Viability of FaDu after 24 h treatment with organic phases of classical extracts of sea buckthorn roots 1 ¬ 8.

	Ethyl acetate	Root 1	Root 2	Root 3	Root 4	Root 5	Root 6	Root 7	Root 8
A	0,6437	0,425	0,4943	0,6501	0,5864	0,7593	0,7837	0,6872	0,4525
B	0,8554	0,7411	0,7956	0,7781	0,7177	0,8403	0,8999	0,8211	0,7094
C	0,93	0,8614	0,8697	0,6941	0,8285	0,8752	0,8561	0,8644	0,7402
D	0,8782	0,8494	0,9028	0,7379	0,8212	0,8843	0,8919	0,9111	0,8423
E	0,9104	0,8166	0,8709	0,8168	0,7937	0,8263	0,8764	0,8842	0,7962
F	0,9027	0,8175	0,8493	0,909	0,754	0,7997	0,8007	0,833	0,81
G	0,7915	0,6868	0,7535	0,8238	0,7498	0,6522	0,745	0,8175	0,6466
H	0,162	0,2209	0,2613	0,4128	0,264	0,4235	0,3803	0,4959	0,1849
mean	0,878	0,795	0,840	0,793	0,777	0,813	0,845	0,855	0,757
Standard deviation	0,050	0,068	0,055	0,075	0,044	0,085	0,060	0,038	0,073
t-value		0,039	0,243	0,047	0,004	0,143	0,327	0,393	0,009
Mean [%]	100,00	90,60	95,70	90,35	88,55	92,59	96,24	97,40	86,27
Standard deviation [%]	5,66	8,52	6,59	9,43	5,66	10,42	7,16	4,41	9,59

Supplementary Table S6 Viability of FaDu after 24 h treatment with aqueous phases of microwave extracts of sea buckthorn roots 1 - 8.

	DMSO	Root 1	Root 2	Root 3	Root 4	Root 5	Root 6	Root 7	Root 8
A	0,1795	0,2386	0,2912	0,3485	0,2486	0,2428	0,2153	0,354	0,2099
B	0,6549	0,4662	0,5479	0,3238	0,343	0,32	0,3212	0,4043	0,3987
C	0,8575	0,8478	0,9009	0,6431	0,4912	0,5371	0,613	0,7423	0,595
D	0,9	0,8297	0,8912	0,8861	0,7941	0,7937	0,8611	0,8606	0,6887
E	0,9483	0,8821	0,9452	0,9611	0,868	0,8894	0,9061	0,8783	0,7448
F	0,9739	0,946	1,0171	0,9388	1,0845	0,8851	0,9921	1,0016	0,7137
G	0,9833	0,9647	0,9445	1,0133	0,964	0,9598	0,9579	0,97	0,6726
H	0,5887	0,7591	0,7759	0,7406	0,4978	0,7599	0,6896	0,8003	0,5277
mean	0,933	0,894	0,940	0,950	0,928	0,882	0,929	0,928	0,683
Standard deviation	0,053	0,059	0,050	0,053	0,126	0,068	0,058	0,069	0,056
t-value		0,311	0,831	0,643	0,945	0,271	0,932	0,909	0,000
Mean [%]	100,00	95,87	100,77	101,85	99,47	94,57	99,65	99,47	73,23
Standard deviation [%]	5,68	6,64	5,29	5,55	13,54	7,72	6,20	7,41	8,23

Supplementary Table S7 Viability of FaDu after 24 h treatment with organic phases of microwave extracts of sea buckthorn roots 1 - 8.

	Ethyl acetate	Root 1	Root 2	Root 3	Root 4	Root 5	Root 6	Root 7	Root 8
A	0,1824	0,2438	0,2957	0,3558	0,2526	0,2478	0,2219	0,3621	0,2127
B	0,6716	0,4749	0,5624	0,3302	0,3449	0,3272	0,3282	0,4115	0,4067
C	0,9053	0,8947	0,9349	0,657	0,4974	0,5507	0,6273	0,7656	0,6105
D	0,9467	0,8739	0,9357	0,917	0,8155	0,8272	0,8867	0,898	0,7195
E	0,9978	0,9323	0,9835	1,0002	0,9162	0,9239	0,9488	0,9202	0,7774
F	1,0183	0,9894	1,0532	0,9737	1,109	0,9366	1,0121	1,0444	0,7551
G	1,03	1,0054	0,9775	1,036	1,0034	0,9921	0,9837	0,9988	0,7094
H	0,6036	0,7844	0,7978	0,7592	0,5246	0,7773	0,7022	0,8218	0,557
mean	0,980	0,939	0,977	0,982	0,961	0,920	0,958	0,925	0,714
Standard deviation	0,052	0,057	0,048	0,050	0,125	0,069	0,054	0,107	0,064
t-value		0,278	0,936	0,953	0,795	0,205	0,563	0,350	0,000
Mean [%]	100,00	95,87	99,73	100,21	98,10	93,91	97,78	94,47	72,92
Standard deviation [%]	5,35	6,12	4,94	5,11	13,01	7,45	5,64	11,57	8,98

Supplementary Table S8 Viability of hMSC after 24 h treatment with aqueous phases of microwave extracts of sea buckthorn roots 1 - 8.

	DMSO	Root 1	Root 2	Root 3	Root 4	Root 5	Root 6	Root 7	Root 8
A	0,185	0,303	0,393	0,316	0,331	0,426	0,379	0,342	0,352
B	0,309	0,368	0,397	0,388	0,387	0,471	0,413	0,403	0,409
C	0,349	0,432	0,457	0,461	0,445	0,514	0,47	0,496	0,465
D	0,419	0,467	0,455	0,49	0,487	0,538	0,503	0,515	0,465
E	0,029	0,47	0,484	0,489	0,475	0,57	0,54	0,536	0,524
F	0,403	0,469	0,434	0,5	0,519	0,547	0,513	0,537	0,512
G	0,447	0,485	0,476	0,546	0,528	0,574	0,539	0,558	0,41
H	0,483	0,511	0,517	0,551	0,507	0,523	0,478	0,559	0,521
mean	0,402	0,457	0,460	0,489	0,515	0,534	0,494	0,478	0,472
Standard deviation	0,064	0,046	0,038	0,055	0,054	0,036	0,045	0,049	0,049
t-value		0,109	0,086	0,025	0,007	0,002	0,016	0,039	0,054
Mean [%]	100,00	113,88	114,52	121,81	128,18	132,91	122,92	119,08	117,58
Standard deviation [%]	15,88	10,04	8,31	11,26	10,52	6,65	9,05	10,27	10,45

Supplementary Table S9 Viability of hMSC after 24 h treatment with organic phases of microwave extracts of sea buckthorn roots 1 - 8.

	Ethyl acetate	Root 1	Root 2	Root 3	Root 4	Root 5	Root 6	Root 7	Root 8
A	0,58	0,553	0,614	0,597	0,596	0,565	0,605	0,54	0,488
B	0,584	0,593	0,552	0,558	0,555	0,531	0,576	0,54	0,486
C	0,595	0,536	0,465	0,539	0,518	0,538	0,584	0,516	0,506
D	0,534	0,528	0,462	0,515	0,52	0,544	0,551	0,491	0,462
E	0,503	0,448	0,481	0,536	0,498	0,513	0,534	0,491	0,456
F	0,463	0,386	0,423	0,478	0,496	0,487	0,49	0,441	0,408
G	0,422	0,419	0,342	0,464	0,452	0,482	0,459	0,366	0,425
H	0,231	0,324	0,382	0,406	0,449	0,448	0,438	0,306	0,37
mean	0,526	0,495	0,477	0,527	0,484	0,523	0,543	0,519	0,462
Standard deviation	0,066	0,077	0,087	0,046	0,062	0,030	0,053	0,046	0,035
t-value		0,434	0,264	0,978	0,242	0,916	0,609	0,834	0,050
Mean [%]	100,00	94,08	90,71	100,16	91,96	99,43	103,21	98,75	87,78
Standard deviation [%]	12,62	15,57	18,34	8,70	12,85	5,83	9,70	8,85	7,66

Supplementary Table S10 Viability of Cal 33 after 24 h treatment with aqueous phases of microwave extracts of sea buckthorn roots 1 - 8.

	DMSO	Root 1	Root 2	Root 3	Root 4	Root 5	Root 6	Root 7	Root 8
A	0,266	0,28	0,326	0,264	0,228	0,217	0,221	0,197	0,174
B	0,535	0,59	0,574	0,54	0,452	0,557	0,413	0,343	0,302
C	0,779	0,734	0,636	0,701	0,561	0,737	0,645	0,492	0,554
D	0,755	0,753	0,662	0,774	0,849	0,802	0,769	0,644	0,651
E	0,751	0,784	0,643	0,812	0,943	0,88	0,751	0,717	0,611
F	0,729	0,92	0,647	0,851	0,871	0,9	0,703	0,755	0,6
G	0,769	0,843	0,632	0,78	0,864	0,802	0,62	0,716	0,5
H	0,349	0,618	0,44	0,472	0,686	0,516	0,445	0,396	0,331
mean	0,757	0,807	0,632	0,784	0,708	0,824	0,698	0,882	0,583
Standard deviation	0,019	0,076	0,030	0,055	0,046	0,066	0,065	0,042	0,058
t-value		0,215	0,000	0,351	0,124	0,083	0,112	0,005	0,002
Mean [%]	100,00	106,63	83,58	103,57	93,58	108,93	92,20	116,54	77,08
Standard deviation [%]	2,52	9,37	4,81	7,07	6,55	8,01	9,28	4,75	9,93

Supplementary Table S11 Viability of Cal-33 after 24 h treatment with aqueous phase of microwave extract of sea buckthorn root 8 and its fractions.

	DMSO	Root 8 (new extract)	fraction 1	fraction 2	fraction 3	fraction 4	fraction 5	fraction 6	fraction 7
A	0,945	0,753	0,742	0,796	0,894	0,928	0,982	0,891	0,831
B	0,871	0,79	0,816	0,735	0,918	0,783	0,954	0,925	0,947
C	0,909	0,74	0,758	0,706	0,899	0,751	0,924	0,885	0,798
D	0,787	0,684	0,768	0,731	0,872	0,731	0,871	0,872	0,926
E	0,865	0,802	0,663	0,827	0,828	0,67	0,797	0,769	0,771
F	0,821	0,758	0,654	0,754	0,749	0,631	0,767	0,792	0,75
G	0,939	0,832	0,632	0,765	0,73	0,571	0,752	0,789	0,73
H	0,744	0,603	0,53	0,745	0,642	0,611	0,591	0,698	0,652
mean	0,860	0,745	0,695	0,757	0,817	0,710	0,830	0,828	0,801
Standard deviation	0,072	0,073	0,093	0,039	0,099	0,115	0,129	0,077	0,099
t-value		0,007	0,002	0,005	0,333	0,009	0,574	0,399	0,193
Mean [%]	100,00	86,64	80,85	88,05	94,93	82,49	96,47	96,22	93,08
Standard deviation [%]	8,37	9,75	13,32	5,09	12,14	16,15	15,60	9,33	12,36

Supplementary Table S12 Cell viability of FaDu after treatment with sucrose, catechin and epicatechin and their mixtures.

	DMSO	sucrose (50 µg/mL)	(+)-Catechin (50 µg/mL)	(-)-Epicatechin (50 µg/mL)	(+)-Catechin (50 µg/mL) + sucrose (50 µg/mL)	(-)-Epicatechin (50 µg/mL) + sucrose (50 µg/mL)	(+)-Catechin (25 µg/mL)	(-)-Epicatechin (25 µg/mL)	(+)-Catechin 25 µg/mL) + sucrose (25 µg/mL)
A	0,594	0,373	0,419	0,485	0,505	0,431	0,233	0,222	0,226
B	0,784	0,793	0,723	0,713	0,818	0,676	0,554	0,545	0,462
C	0,824	0,946	0,949	0,856	0,981	0,734	0,605	1,002	0,879
D	0,831	0,962	0,968	0,839	0,97	0,886	1,026	1,071	1,015
E	0,935	0,922	0,961	0,863	0,899	0,877	0,931	0,993	0,994
F	0,958	0,936	0,939	0,871	0,896	0,923	0,933	0,95	0,973
G	0,99	0,958	0,927	0,893	0,875	0,881	0,943	0,931	0,758
H	0,541	0,74	0,742	0,742	0,604	0,721	0,685	0,473	0,392
mean	0,887	0,920	0,911	0,839	0,907	0,860	0,958	0,989	0,924
Standard deviation	0,084	0,064	0,093	0,064	0,061	0,073	0,045	0,054	0,106
t-value		0,470	0,648	0,297	0,657	0,586	0,124	0,039	0,549
Mean [%]	100,00	103,66	102,72	94,61	102,20	96,98	108,03	111,54	104,15
Standard deviation [%]	9,52	6,92	10,25	7,66	6,72	8,47	4,75	5,49	11,50

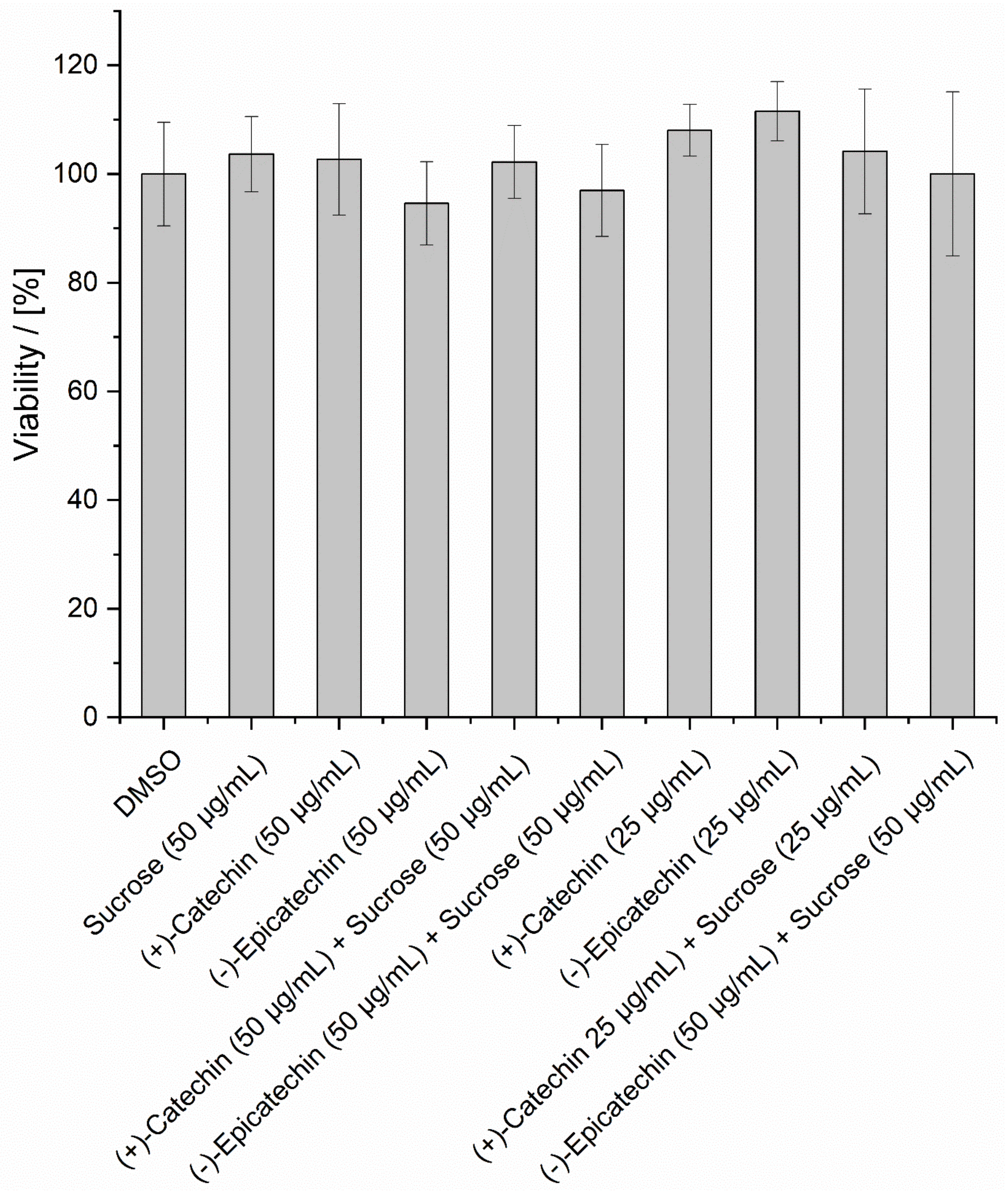

Supplementary Figure S24 Cell viability after treatment of cells of cancer cell lines FaDu with sucrose, catechin and epicatechin and their mixtures. Mean ± SD, n = 4 – 6.

7. Flow Cytometry

Supplementary Table S13 Number of cells of the cell line FaDu after 24 h treatment with the aqueous phase of the microwave extract of sea buckthorn root 8 and its fractions in the respective cell cycle phases.

	Sum	G1	S	G2	Sub G1
DMSO	10136	4566	1023	1908	451
	10251	4522	1092	1980	239
Root 8	10324	2745	1315	2473	291
	10195	3266	1537	2475	285
Fraction 1	10218	4852	357	2432	465
	10090	4204	714	2564	547
	10123	4162	553	2597	565
Fraction 4	7965	3021	1003	1600	912
	10247	2993	1146	2152	929

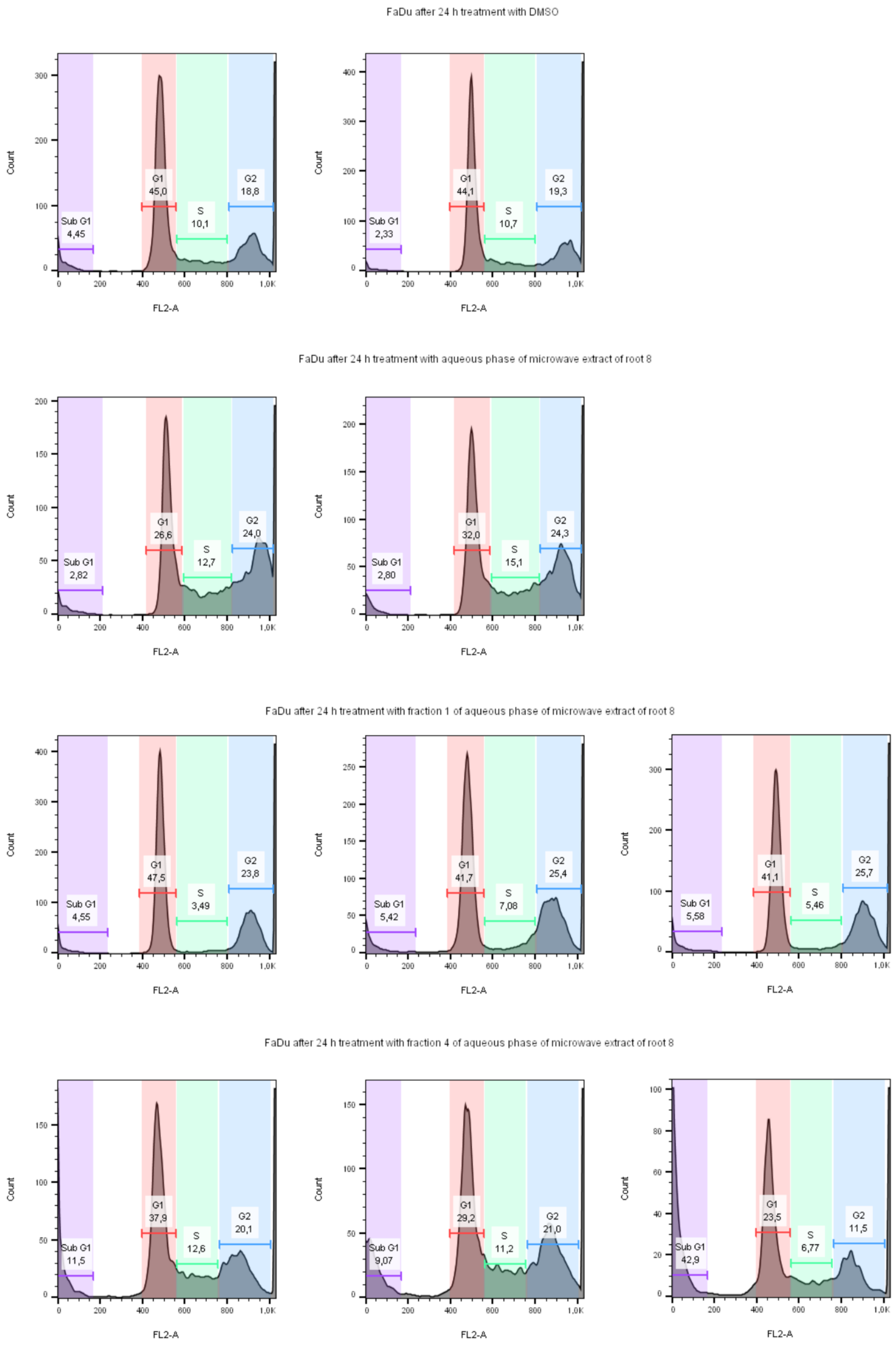

Supplementary Figure S25 Cell cycle of FaDu after 24 h treatment.

Supplementary Table S14 Number of cells of the cell line Cal 33 after 24 h treatment with the aqueous phase of the microwave extract of sea buckthorn root 8 and its fractions in the respective cell cycle phases.

	Sum	G1	S	G2	Sub G1
DMSO	20971	5967	1561	2946	2009
	17130	5301	1399	2442	1339
	13995	4289	1070	2254	793
Root 8	17340	4995	1995	2555	1609
	19230	5555	1772	2517	2823
	5085	1522	282	762	835
Fraction 1	17340	4995	1995	2555	1609
	19230	5555	1772	2517	2823
	5085	1522	282	762	835
Fraction 4	16350	4202	905	2265	1563
	17325	5107	1331	2681	2063
	15600	3965	1183	2556	1462

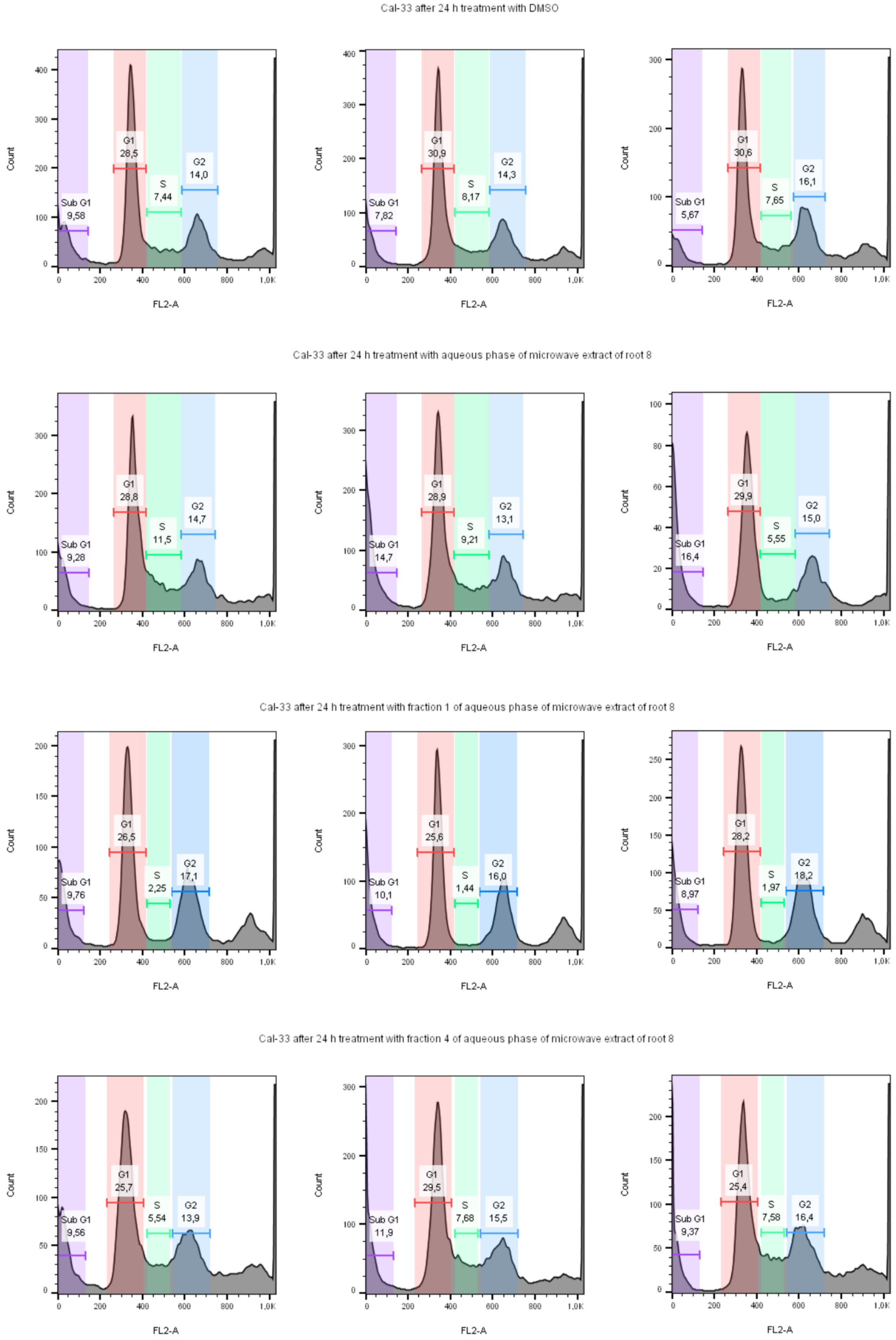

Supplementary Figure S26 Cell cycle of Cal 33 after 24 h treatment.

Supplementary Table S15 Number of cells of the cell line FaDu after 48 h treatment with the aqueous phase of the microwave extract of sea buckthorn root 8 and its fractions in the respective cell cycle phases.

	Sum	G1	S	G2	Sub G1
DMSO	10507	3997	1694	1877	1231
	10388	4031	1606	1880	1344
	10547	3648	1566	1901	1648
Root 8	10675	3238	1619	1572	1511
	10653	3152	1520	1608	1702
	10678	2901	1335	1568	2212
Fraction 1	10338	3681	662	810	3825
	10821	4572	705	1325	2432
	10471	2438	786	1129	4712
Fraction 4	10897	3289	1346	1684	2291
	11035	3886	1588	1845	1239
	10840	3349	1542	1902	1541

FaDu after 48 h treatment with DMSO

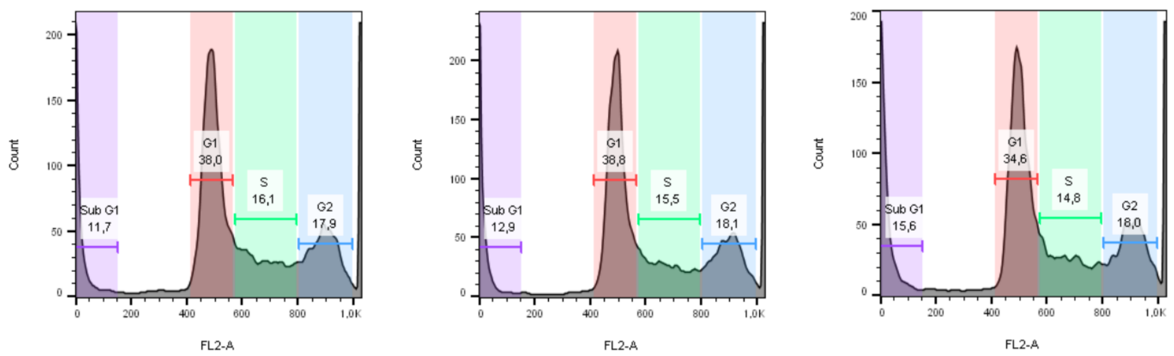

FaDu after 48 h treatment with aqueous phaseof microwave extract of root 8

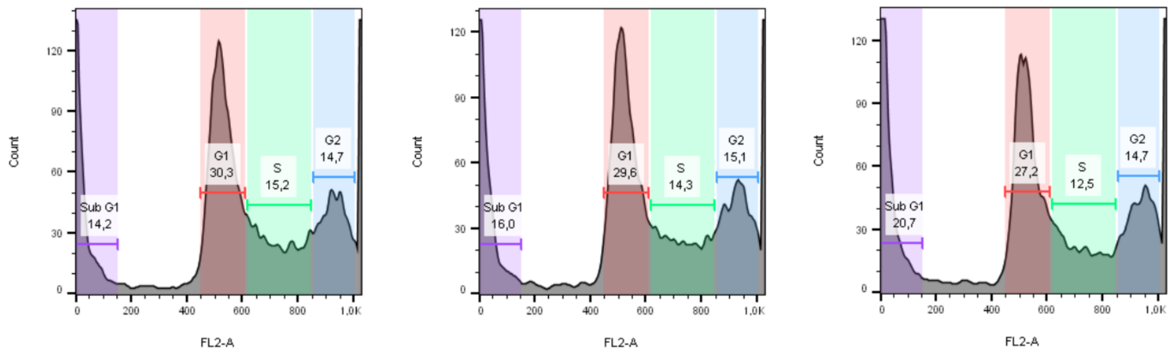

FaDu after 48 h treatment with fraction 1 of aqueous phase of microwave extract of root 8

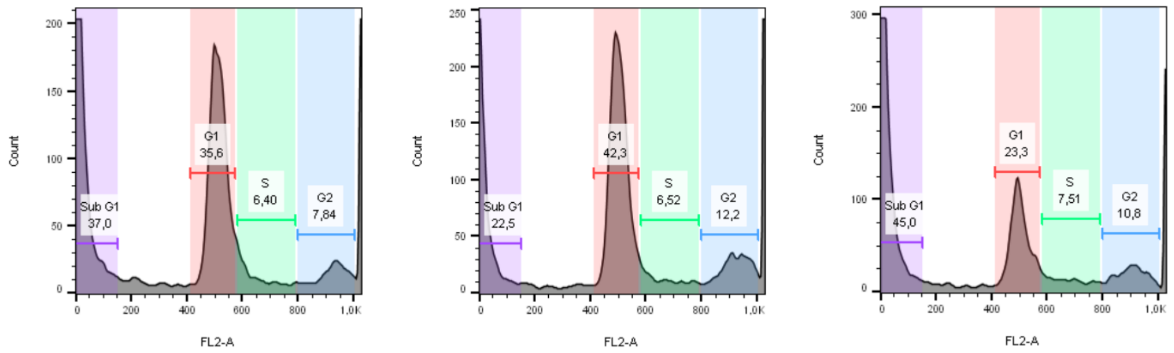

FaDu after 48 h treatment with fraction 4 of aqueous phase of microwave extract of root 8

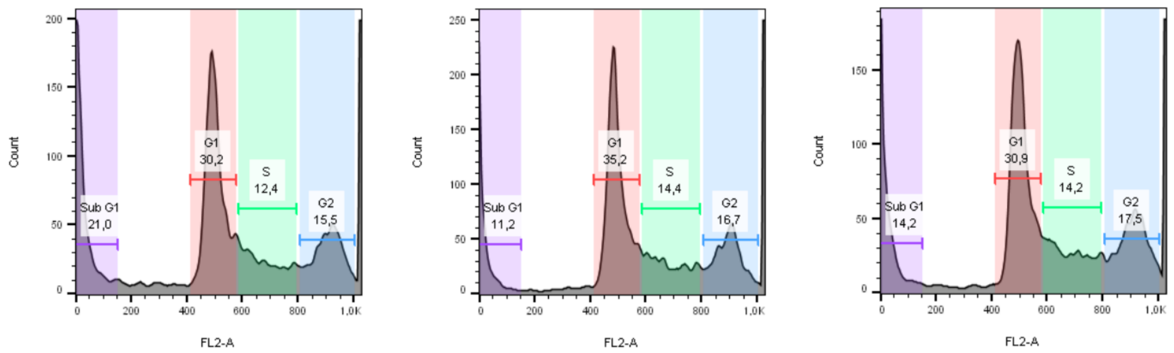

Supplementary Figure S27 FaDu after 48 h treatment.

Supplementary Table S16 Number of cells of the cell line FaDu after 72 h treatment with the aqueous phase of the microwave extract of sea buckthorn root 8 and its fractions in the respective cell cycle phases.

	Sum	G1	S	G2	Sub G1
DMSO	10269	6819	441	1069	545
	10214	7228	337	1074	636
	10224	6974	467	1119	472
Root 8	10205	6452	634	1473	464
	10156	6941	646	1289	465
	10137	6399	820	1532	402
Fraction 1	10121	6597	609	1125	715
	10402	6694	431	1395	546
	10212	7032	459	1175	651
Fraction 4	10368	7256	474	1041	633
	10532	6654	457	1454	605
	10527	6850	416	1484	532

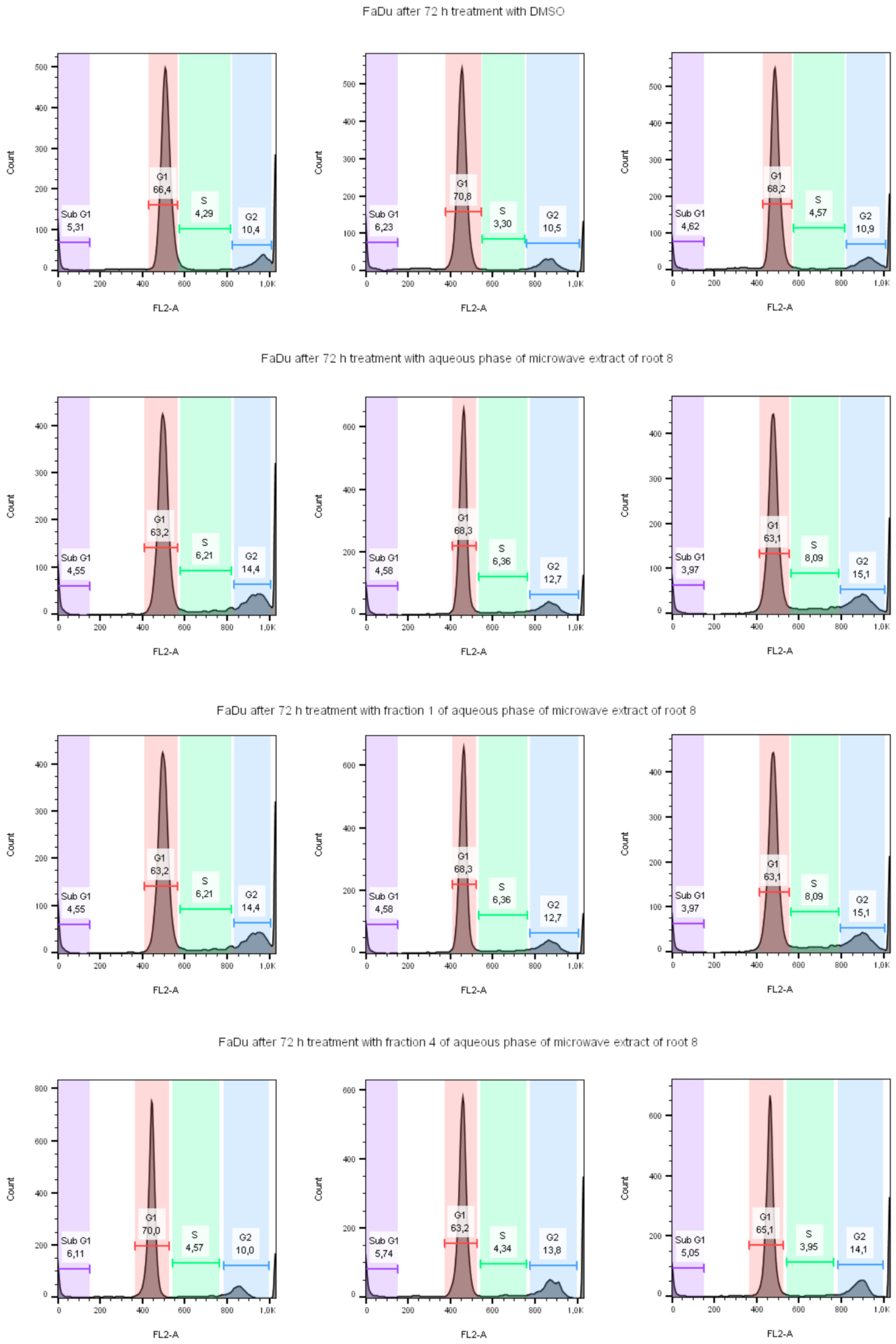

Supplementary Figure S28 FaDu after 72 h treatment.

9. ECIS

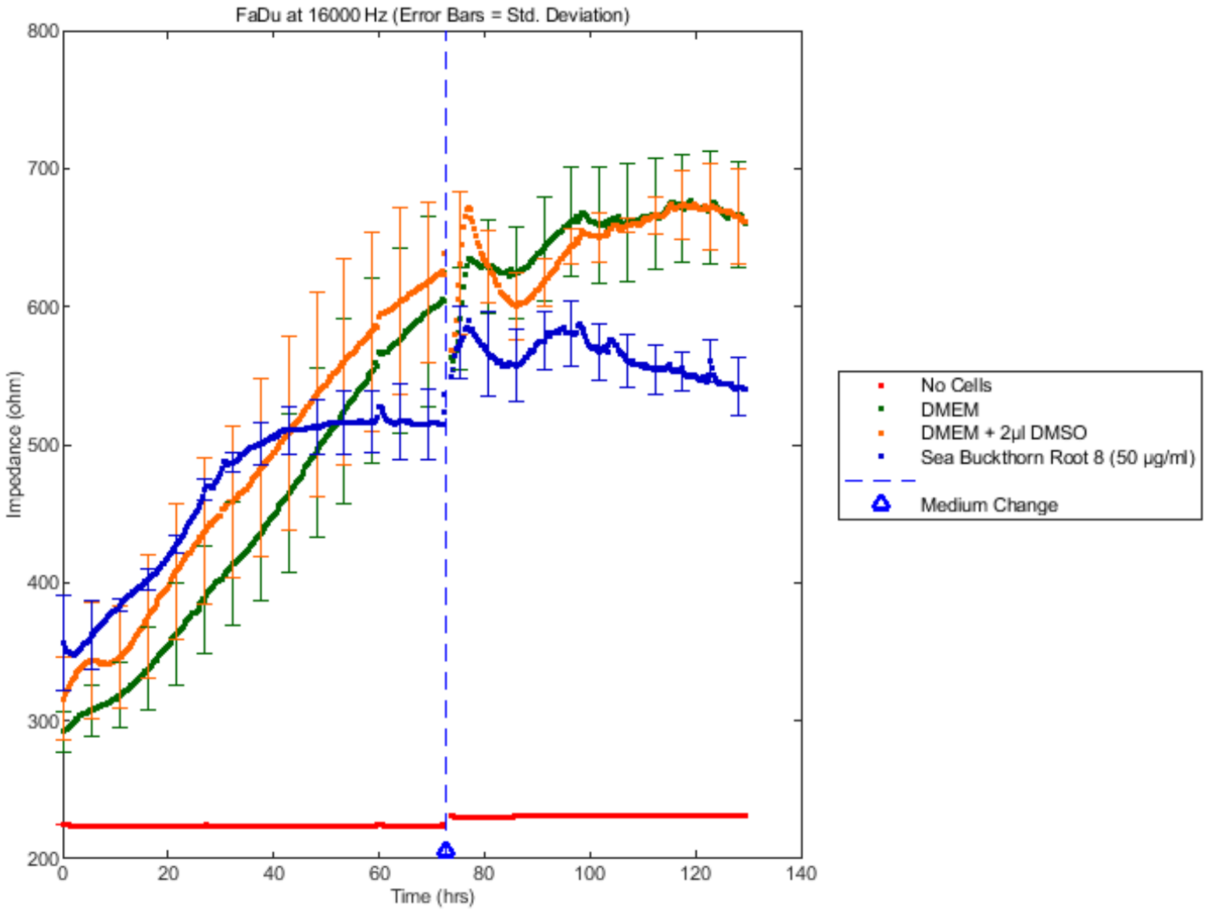

Supplementary Figure S29 Non-normalized impedance of FaDu after treatment with DMSO and the aqueous phase of the microwave extract of sea buckthorn root 8 measured at 16000 Hz.
